# Analysis of immune-related signatures of lung adenocarcinoma identified two distinct subtypes: implications for immune checkpoint blockade therapy

**DOI:** 10.18632/aging.102814

**Published:** 2020-02-24

**Authors:** Qinghua Wang, Meiling Li, Meng Yang, Yichen Yang, Fengju Song, Wei Zhang, Xiangchun Li, Kexin Chen

**Affiliations:** 1 Department of Epidemiology and Biostatistics, National Clinical Research Center for Cancer, Key Laboratory of Molecular Cancer Epidemiology of Tianjin, Tianjin Medical University Cancer Institute and Hospital, Tianjin 300060, China; 2 Tianjin Cancer Institute, National Clinical Research Center for Cancer, Key Laboratory of Cancer Prevention and Therapy of Tianjin, Tianjin Medical University Cancer Institute and Hospital, Tianjin 300060, China; 3 Wake Forest Baptist Comprehensive Cancer Center, Wake Forest Baptist Medical Center, Winston-Salem, NC 27157, USA

**Keywords:** lung adenocarcinoma, immune subtyping, immunotherapy, prognosis

## Abstract

Immune checkpoint blockade (ICB) therapies have revolutionized the treatment of human cancers including lung adenocarcinoma (LUAD). However, our understanding of the immune subtyping of LUAD and its association with clinical response of immune checkpoint inhibitor remains incomplete. Here we performed molecular subtyping and association analysis of LUAD from the Cancer Genome Atlas (TCGA) and validated findings from TCGA cohort in 9 independent validation cohorts. We conducted consensus molecular subtyping with nonnegative matrix factorization (NMF). Potential response of ICB therapy was estimated with Tumor Immune Dysfunction and Exclusion (TIDE) algorithm. We identified 2 distinct subtypes of LUAD in TCGA cohort that were characterized by significantly different survival outcomes (i.e., high- and low-risk subtypes). The high-risk subtype was featured by lower TIDE score, upregulation of programmed death-ligand 1 (*PD-L1*) expression, and higher tumor mutation burden (TMB). The high-risk subtype also harbored significantly elevated cell cycle modulators CDK4/CDK6 and *TP53* mutation. These observations were validated in 9 independent LUAD cohorts. Our findings suggest that immune checkpoint blockade therapy may be efficacious for high-risk subtype of LUAD patients.

## INTRODUCTION

Lung adenocarcinoma (LUAD) is a leading cause of cancer related death worldwide and accounts for about 40% of lung cancer patients [1]. Over the last decade, treatment regimens that target epidermal growth factor receptor and anaplastic lymphoma kinase have only benefitted a small fraction of LUAD patients [2, 3]. Clinically defined molecular subtyping of LUAD is in urgent need for precise treatment.

Programmed cell death protein 1/programmed cell death-ligand 1 (PD-1/PD-L1) axis is a critical immune checkpoint pathway that could downregulate response of immune system in lung cancer [4]. Enhanced PD-L1 expression level on tumor cells or tumor infiltrating lymphocytes leads to T cell exhaustion [5], therefore decreasing tumor-specific immune capacity and promoting tumor proliferation [6, 7]. Immune checkpoint blockade (ICB) therapy aimed at PD-1/PD-L1 could repress the negative regulatory signaling and unleash T cell from exhausted status [7]. ICB therapy can reverse the immunosuppressive microenvironment by decreasing the potential of tumor immune escape, therefore, resulting in noteworthy improvement of prognosis [8, 9]. High expression level of PD-L1 was approved by The Food and Drug Administration (FDA) as a biomarker to receive pembrolizumab therapy in non-small-cell lung cancer (NSCLC) [8].

Tumor mutation burden (TMB) is an indicator implicated in better response to ICB treatment, which is independent of PD-L1 expression level [10, 11]. An integrated analysis of 27 cancer types reported positive association between TMB and benefit of ICB treatment [12]. In present clinical practice, the proportion of patients benefitted from ICB therapy remains low. Therefore, the development of new biomarkers to select patient for ICB response rate is definitely required [13, 14].

Tumor Immune Dysfunction and Exclusion (TIDE) algorithm is a computational method using gene expression profile to predict the ICB response in NSCLC and melanoma [15]. TIDE uses a set of gene expression markers to estimate 2 distinct mechanisms of tumor immune evasion, including dysfunction of tumor infiltration cytotoxic T lymphocytes (CTL) and exclusion of CTL by immunosuppressive factors. Patients with higher TIDE score have a higher chance of antitumor immune escape, thus exhibiting lower response rate of ICB treatment [15]. The TIDE score was shown to have a higher accuracy than PD-L1 expression level and TMB in predicting survival outcome of cancer patients treated with ICB agents [15–18]. Several recent studies have reported its utility in predicting or evaluating the ICB therapy efficacy [18–22].

In this study, we performed consensus clustering analysis for LUAD based on immune gene expression signatures and identified 2 subtypes. These 2 subtypes were characterized by significantly different survival outcomes (i.e., low- versus high-risk subtypes), TIDE scores, PD-L1 expression, TMB and enrichment of cell cycle signaling. The high-risk subtype is presumably efficacious towards ICB treatment.

## RESULTS

### Identification of LUAD subtypes with prognostic significance

**Figure 1:**
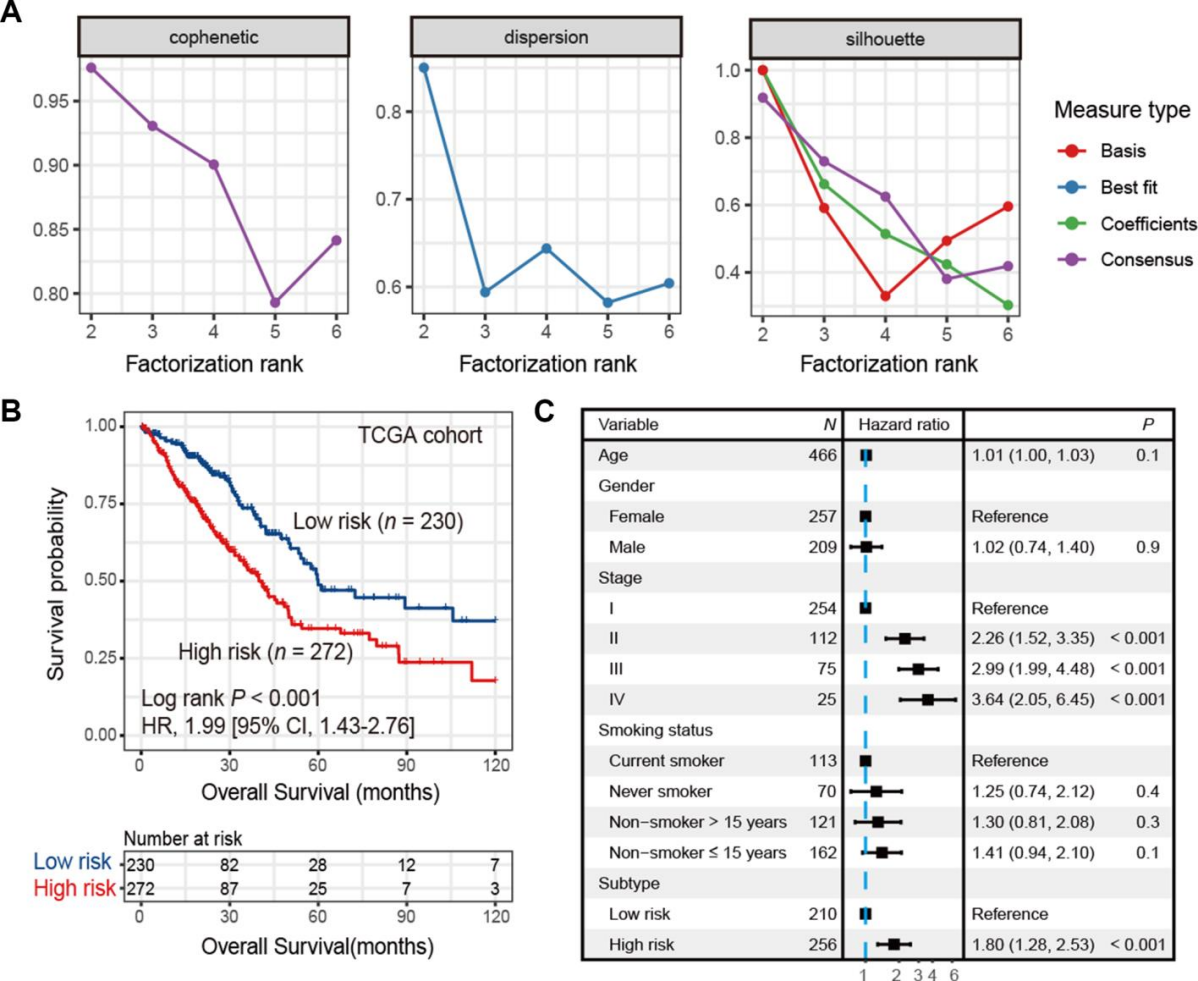
**Identification of high-risk and low-risk subtypes of LUAD in TCGA cohort by consensus clustering.** (**A**) The relationship between cophenetic, dispersion and silhouette coefficients with respect to number of clusters. (**B**) Kaplan-Meier survival plot of the high-risk versus the low-risk subtype. (**C**) Forest plot representation of multivariate Cox model depicted association between overall survival and LUAD subtypes with other clinical factors taken into account.

A list of 2,995 immune-related genes (See Materials and Methods) was compiled, 433 of which were significantly associated with overall survival (all FDR < 0.05; Supplementary Table 1) in the TCGA LUAD cohort (*n* = 502). Using this 433-gene panel, we identified 2 distinct LUAD subtypes (Figure 1A and Supplementary Figure 1) by performing consensus clustering analysis. These 2 subtypes were significantly different in survival (HR: 1.99, 95% CI: 1.43-2.76, Log rank *P* < 0.001; Figure 1B) and we referred them as high-risk and low-risk subtypes. After adjusting for age, gender, stage and smoking status, this association remained statistically significant (HR: 1.80, 95% CI: 1.28-2.53, *P* < 0.001; Figure 1C).

**Figure 2:**
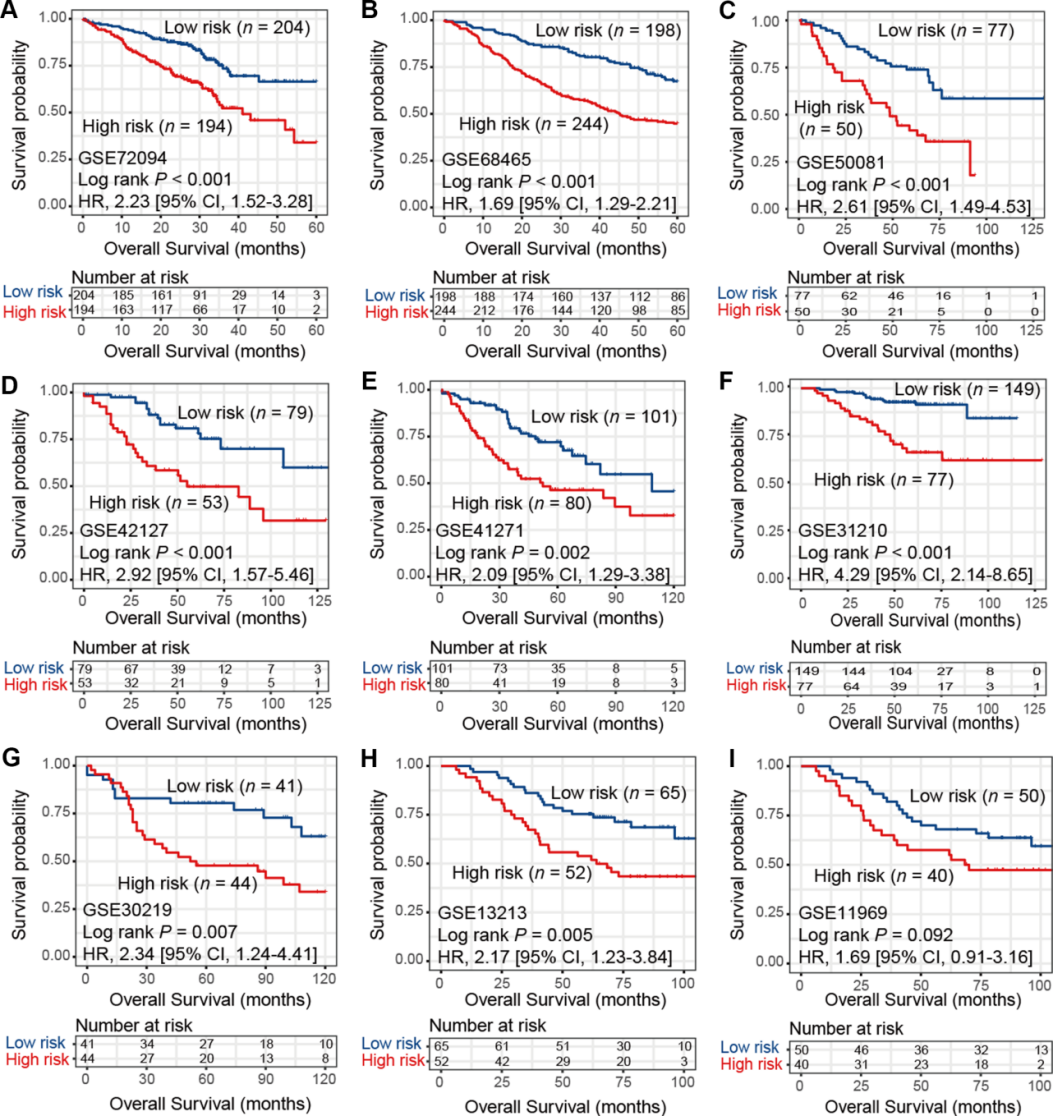
Kaplan-Meier plots of high-risk and low-risk subtypes of LUAD in 9 validation cohorts of (**A**) GSE72094, (**B**) GSE68465, (**C**) GSE50081, (**D**) GSE42127, (**E**) GSE41271, (**F**) GSE31210, (**G**) GSE30219, (**H**) GSE13213, and (**I**) GSE11969.

To verify the findings in TCGA LUAD cohort, we applied consensus clustering analysis using the aforementioned 433 immune-related genes and performed survival analysis in 9 additional independent LUAD cohorts. Our analysis showed that these 2 distinct subtypes identified in TCGA LUAD cohort were also identified in these 9 validation datasets, in that high-risk group was associated with worse survival outcome (HR range: 1.69 [95% CI: 1.29-2.21, Log rank *P* < 0.001] to 4.29 [95% CI: 2.14-8.65, Log rank *P* < 0.001]; Figure 2A–2I). These associations remained statistically significant after controlling for other confounding factors (Supplementary Figure 2A–2I). Result of another LUAD dataset GSE81089 (*n* = 108) showed a consistent trend although did not reach statistical significance (HR: 1.59, 95% CI: 0.89-2.85, Log rank *P* = 0.11; Supplementary Figure 3A). No significant association was observed for lung squamous cell carcinoma from TCGA dataset (HR: 1.03, 95% CI: 0.78-1.35, Log rank *P* = 0.86; Supplementary Figure 3B).

We obtained an accuracy of 0.947 with 126 genes (Supplementary Figure 4A and Supplementary Table 2) by applying recursive feature elimination to reduce the number of genes. The prognostic significance of high-risk versus low-risk subtypes was maintained using these 126 genes in TCGA and 9 validation cohorts (HR range: 1.84 [95% CI: 1.37-2.48, Log rank *P* < 0.001] to 5.99 [95% CI: 2.72-12.23, Log rank *P* < 0.001]; Supplementary Figure 4B–4K).

### Predictive ICB response of identified LUAD subtypes

**Figure 3:**
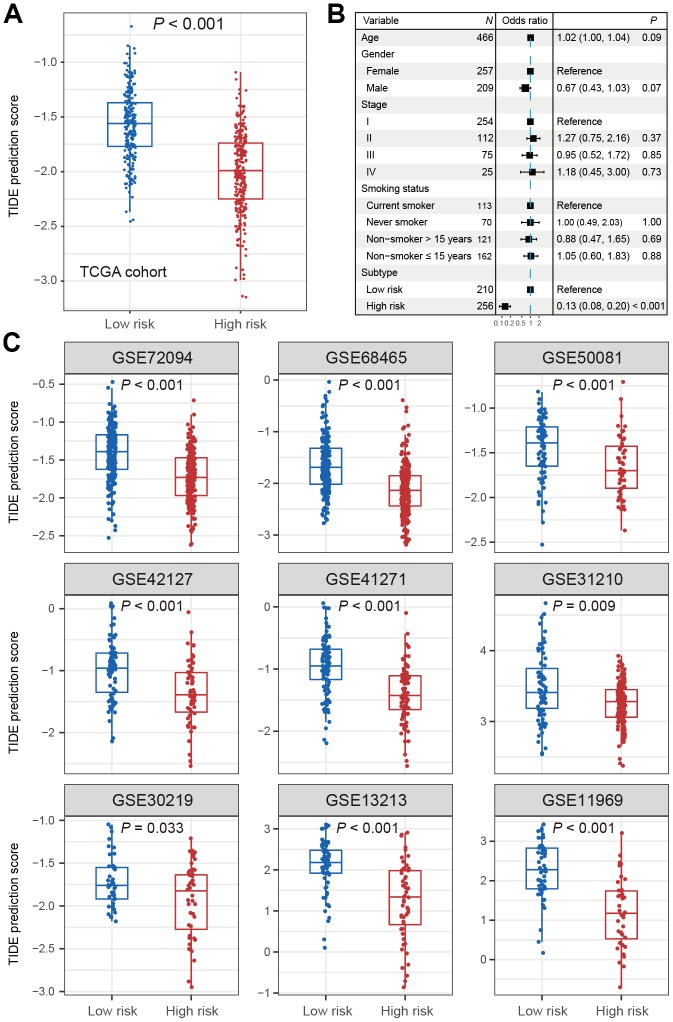
**Distribution of TIDE scores in high-risk subtype of LUAD versus low-risk subtype of LUAD.** (**A**) Boxplot representation of TIDE scores in the high-risk group versus low-risk group in TCGA LUAD cohort. (**B**) Forest plot representation of multivariate model with adjustment for confounding factors in TCGA cohort. (**C**) Distribution of TIDE scores in 9 independent validation cohorts.

In the TCGA LUAD cohort, the TIDE score was significantly lower in high-risk subtype compared with low-risk subtype (Wilcoxon rank-sum test, *P* < 0.001; Figure 3A). The difference remained statistically significant after adjusting for age, gender, stage and smoking status (OR: 0.13, 95% CI: 0.08-0.20, *P* < 0.001; Figure 3B). This association was verified in 9 independent cohorts using univariate analysis (Wilcoxon rank-sum test, *P* < 0.05; Figure 3C), and 8 of these 9 cohorts showed the same association through multivariate logistic model (*P* < 0.001; Supplementary Figure 5A–5I). These discoveries suggested that patients of high-risk subtype may be more sensitive to ICB therapy as judged by the TIDE score.

### Differences of *PD-L1* expression and TMB between 2 LUAD subtypes

**Figure 4:**
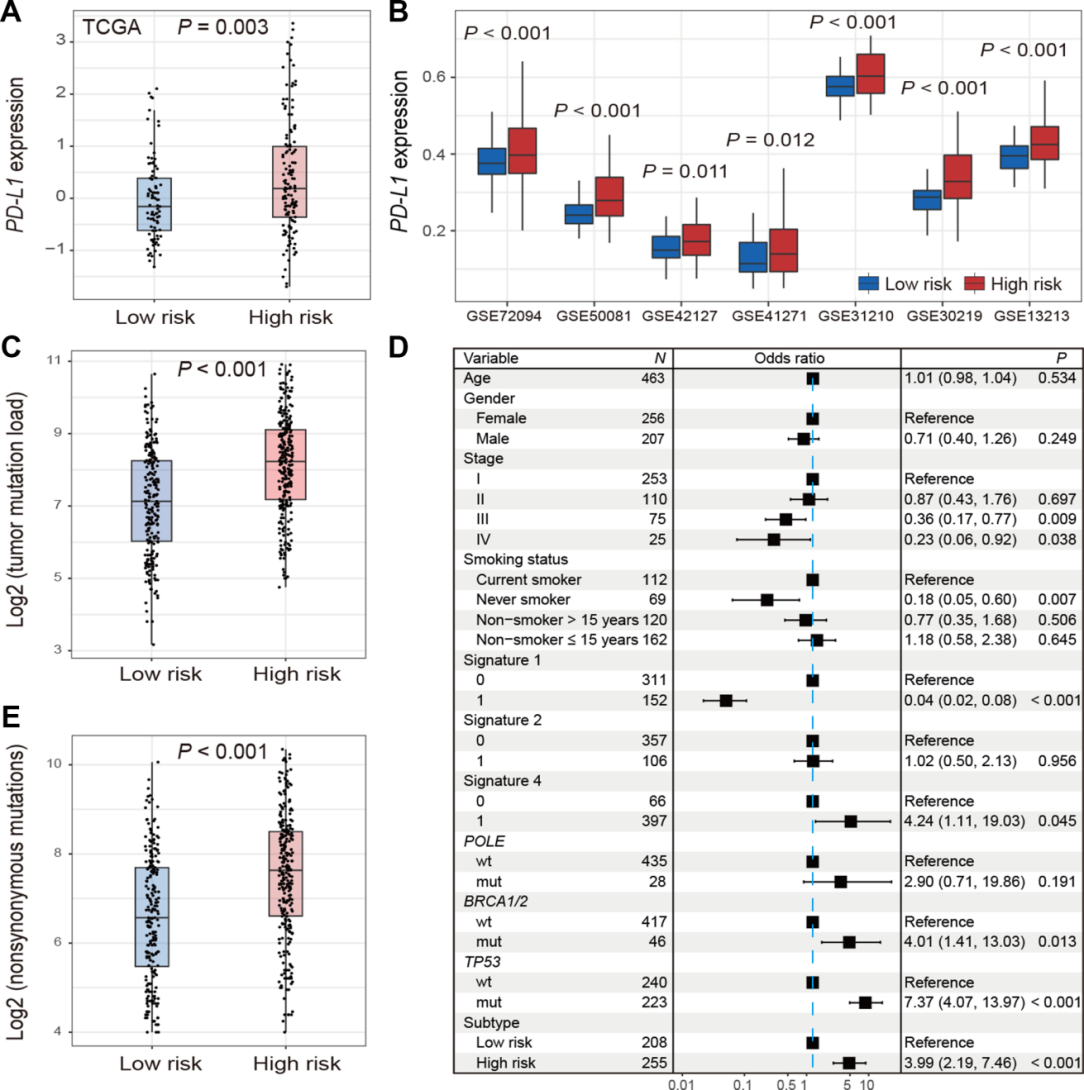
**Distribution of *PD-L1* expression and tumor mutation burden and their associations with the high-/low-risk subtypes of LUAD.** (**A**, **B**) Difference in the *PD-L1* expression in TCGA and validation cohorts stratified by high-/low-risk subtypes of LUAD. (**C**–**E**) Distribution and association of mutation burden in the high-risk group versus low-risk group.

The expression of *PD-L1* was significantly higher in high-risk group versus low-risk group in TCGA (Wilcoxon rank-sum test, *P* = 0.003; Figure 4A). Consistent association was also observed in 7 of 9 validation cohorts (Wilcoxon rank-sum test, *P* < 0.05; Figure 4B). We could not validate this association in the other 2 cohorts (GSE68465 and GSE11969) due to the lack of *PD-L1* probes on the expression chip used.

Patients in high-risk subtype had a significantly higher mutation load in TCGA LUAD samples (Wilcoxon rank-sum test, *P* < 0.001; Figure 4C). Associations between high-risk subtype and TMB remained statistically significant (OR: 3.99, 95% CI: 2.19-7.46, *P* < 0.001; Figure 4D) after taking into account mutational signatures implicated in age-related deamination of 5-methylcytosine (signature 1), overactivity of mRNA-editing enzyme APOBEC (signature 2) and tobacco smoking-related C>A mutations (signature 4) (Supplementary Figure 6), and mutations in genes related to DNA damage repair (DDR) such as *POLE*, *BRCA1/2*, and *TP53*. Additionally, we found that high-risk subtype harbored a significantly higher nonsynonymous mutation load (Wilcoxon rank-sum test, *P* < 0.001; Figure 4E).

In TCGA LUAD cohort, association of TMB with survival was not significant. Associations of TIDE score and *PD-L1* expression with survival were not significant across TCGA LUAD cohort and the 9 validation cohorts (Supplementary Figure 7).

### Functional characterization of the high-risk subtype

**Figure 5:**
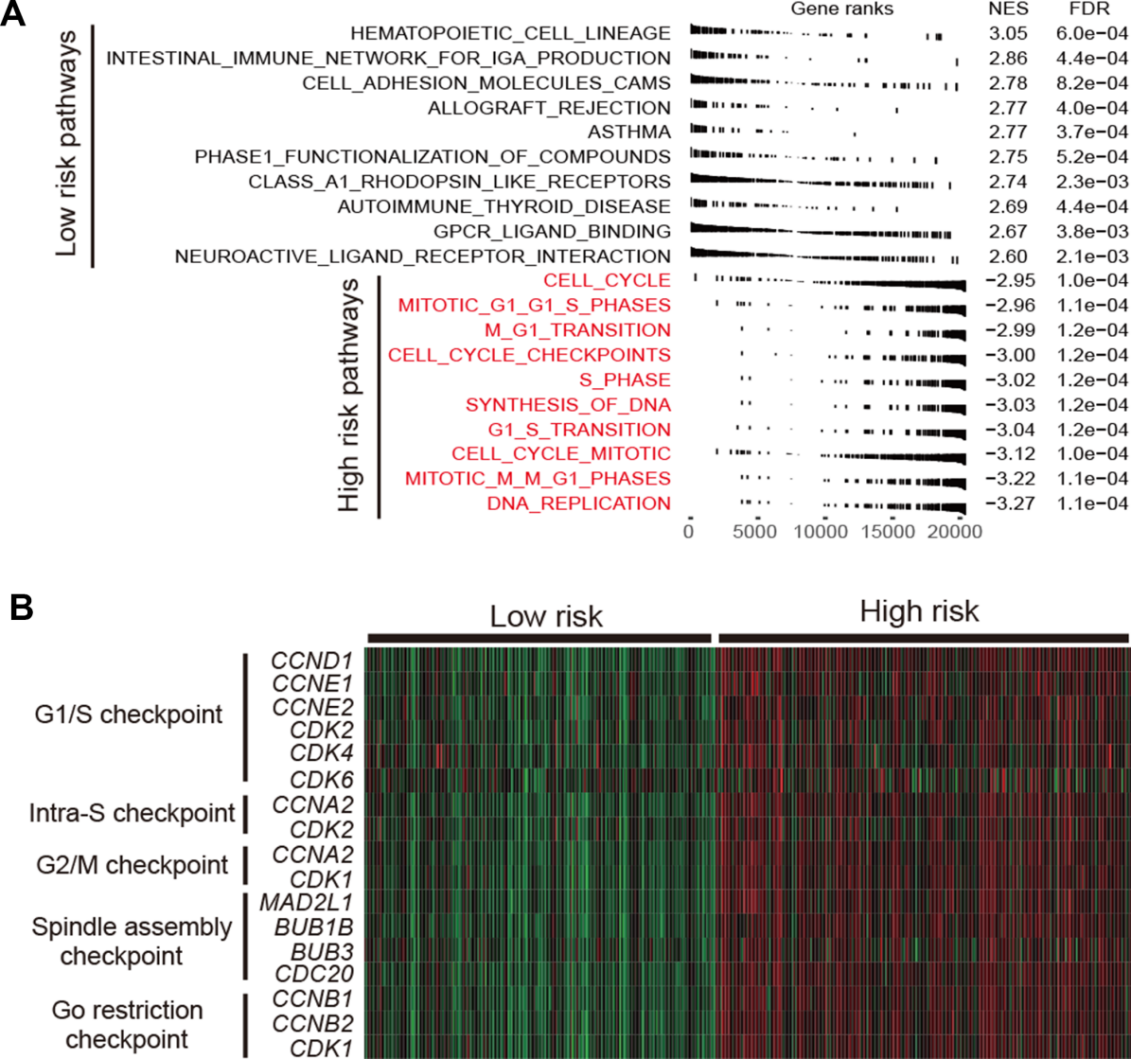
**Functional annotation in high-/low-risk subtypes of LUAD.** (**A**) Dysregulation of signaling pathways stratified by identified LUAD subtypes. (**B**) Expression profiles of cell cycle checkpoint markers in the high-risk group versus low-risk group.

Cell cycle relevant signaling pathways were frequently activated in high-risk group as compared with low-risk group (FDR < 0.001; Figure 5A). Molecular markers involved in cell cycle checkpoint were significantly upregulated in the high-risk subtype (Wilcoxon rank-sum test, *P* < 0.001; Figure 5B). The cell cycle checkpoint markers analyzed include *CCND1*, *CCNE1*, *CDK2*, *CDK4* and *CDK6* in G1/S checkpoint, and *CCNA2* and *CDK1* in G2/M checkpoint. Previous studies reported that CDK4 and CDK6 inhibitors could enhance T cell activity [23] and reverse the T cell exclusion signature [24] to obtain better ICB treatment response.

### Mutation patterns of SMGs in relation to LUAD subtypes

**Figure 6:**
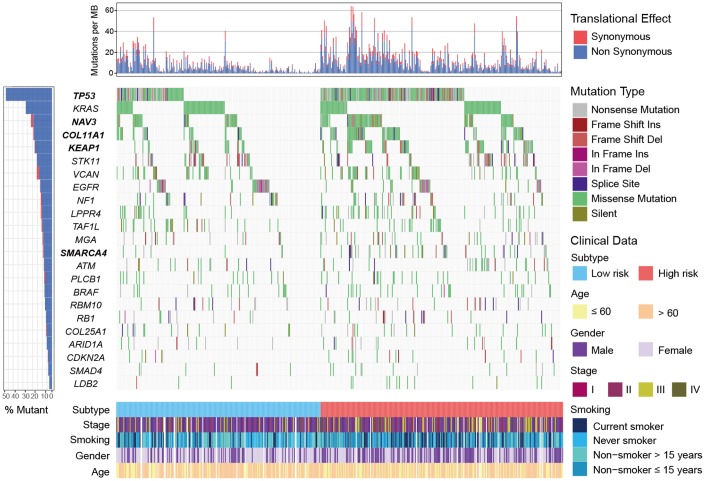
**Mutational landscape of SMGs in TCGA LUAD cohort stratified by high-/low-risk subtypes.** SMGs with significantly different mutation rate were highlighted in bold.

We identified 23 significantly mutated genes (SMGs) in TCGA LUAD cohort (Supplementary Figure 8). *TP53*, *NAV3*, *COL11A1*, *KEAP1* and *SMARCA4* were more frequently mutated in high-risk subtype (Fisher exact test, OR > 1, *P* < 0.05; Figure 6). Association of these SMGs with high-risk subtype remained significant after including age, gender, stage and smoking status (OR > 1, *P* < 0.01; Supplementary Figure 9). Higher mutation frequency of *TP53* in high-risk subtype was observed in 3 validation cohorts that had *TP53* mutation data (OR > 1, *P* < 0.01; Supplementary Figure 10). Mutation data of *NAV3, COL11A1, KEAP1* and *SMARCA4* of the aforementioned 3 validation cohorts was unavailable. In addition, we found that patients in high-risk subtype were more likely to be male, current smoker, and at advanced clinical stage (chi-square test, *P* < 0.001; Supplementary Table 3).

## DISCUSSION

We uncovered 2 prognostic subtypes of LUAD by analyzing 2,300 LUAD samples from TCGA LUAD cohort and 9 independent validation cohorts. The high-risk subtype has significantly lower TIDE score, higher *PD-L1* expression and higher TMB. Pathway analysis suggested that high-risk group was featured by activated cell cycle signaling. *TP53* mutation was more frequently mutated in the high-risk subtype.

We observed that TIDE score was significantly lower in high-risk subtype than low-risk subtype via univariate regression analysis in TCGA LUAD cohort and 9 validation cohorts. The difference remained statistically significant in multivariate analysis among these cohorts except GSE30219, this is probably due to smaller sample size of GSE30219 cohort (*n* = 85) as compared with the other LUAD cohorts. These findings suggested that patients of high-risk subtype may be more sensitive to ICB treatment

Based on the KEYNOTE-001 clinical trial, PD-L1 high expression was an essential condition for the use of pembrolizumab in NSCLC [8, 25]. Several studies have reported that PD-L1 high expression was correlated to elevated response rate and survival benefit in ICB therapy of NSCLC, chronic lymphocytic leukemia and urothelial cancer [26–28]. However, in the clinical trial CHECKMATE-032 that enrolled patients with urothelial cancer, no significant difference was observed in response rate between PD-L1 positive and negative subgroups [29]. This suggests that our understanding of association between PD-L1 and ICB response is far from complete and that new markers associated with ICB response are needed. A possible explanation for this inconsistency is attributed to different criterion used for evaluating high and low PD-L1 expression [30]. TMB is emerging as a potential biomarker for predicting response of ICB therapy. Three clinical trials including KEYNOTE-001, CHECKMATE-026 and CHECKMATE-227 showed that patients had high TMB could benefit more from ICB therapy in NSCLC [31–33]. In our study, the high-risk subtype had significantly higher *PD-L1* expression and TMB, suggesting a greater potential of ICB therapy response.

Consistent with our findings, Seo et al. identified the immune-defective and immune-competent subtypes in LUAD, and the immune-competent subtype was characterized by activated microenvironment and elevated expression of immune checkpoint genes [34]. In our study, we observed a significant prognostic difference between the high-risk and low-risk subtypes. However, a survival difference was not observed for the 2 subtypes proposed by Seo et al. A recent study from Song et al. also reported identification of the high-risk and low-risk subtypes of LUAD [35]. The high-risk subtype in this study exhibited worse survival outcome and a higher tumor mutation load, which supports our result that the high-risk subtype may be more responsive to immune checkpoint therapy because of the higher tumor mutation load. The prognostic significance of high-risk and low-risk subtypes from our study was validated in GSE31210, a validation dataset used in Song et al. research, whereas subtypes proposed by Song et al. was not significant. Taken together, the gene panels used in our study for molecular subtyping are more generalizable and robust given that we included more validation sets (n=9) as compared with 4 validation cohorts used by Song et al.

Cell cycle relevant pathways and checkpoint markers contributed mostly to the worse prognosis of high-risk subtype were significantly upregulated. These observations suggested the high-risk patients may be suitable to receive cell cycle inhibitors. Previous studies showed that CDK4 and CDK6 inhibitors enhanced the response of ICB in mouse models due to their ability to elevate expression of endogenous retroviruses [36] that was associated with T cell activity [23] and ICB response [37]. Another study reported that the T cell exclusion signature was predictive of poor ICB response, however, CDK4 and CDK6 inhibitors could reverse this signature to get a better response in *in vitro* experiments in melanoma [24]. The combination therapy of cell cycle inhibitors and ICB agents may be more effective for the high-risk patients.

*TP53* was frequently mutated in the high-risk subtype and its mutation was reported to be associated with poorer prognosis [38, 39]. Patients harbored mutations of *TP53* had a higher TMB owing to loss of DNA repair function (Figure 4D). Recent study reported that *TP53* mutations significantly induced the expression of immune checkpoints and activated T-effector and interferon-γ signature in LUAD, suggesting *TP53* mutation patients would be more responsive to checkpoint blockade [40]. Patients of high-risk subtype were more likely to be current smokers. Higher response rate of ICB treatment was observed in smokers of NSCLC [41], typically due to high mutation burden generated by the mutagenic effects of cigarette smoke [42]. These 2 factors may underlie the response to ICB therapy for patients of high-risk group.

There are several limitations in this study. Firstly, gene expression data used in our study was from different platforms, this difference may introduce bias in the analysis procedure. Secondly, results derived from TCGA LUAD mutational landscape were not validated in independent datasets due to the unavailability of mutation data in validation sets. Thirdly, we lacked an in-house validation set.

In summary, we identified 2 prognostically and clinically relevant subtypes of LUAD. Molecular markers suggest that patients from the high-risk subtype may be more responsive to ICB therapy, which needs to be tested in future clinical trials.

## MATERIALS AND METHODS

### Collection of genomic data

We collected gene expression profiles of 502 LUAD samples from TCGA-LUAD cohort (https://gdc.cancer.gov) and 1,798 samples from 9 cohorts in Gene Expression Omnibus (GEO) repository (https://www.ncbi.nlm.nih.gov/geo/) (Supplementary Table 4). In total, we obtained 2,300 LUAD samples from 10 independent cohorts (Supplementary Table 5). We also collected clinical data for each cohort. All gene expression data were uniformly normalized. For genes with multiple probes, their expression levels were calculated as the mean expression level of these probes. Only TCGA cohort contained genomic mutation data used in our study. A flow chart to depict the study design was shown in Supplementary Figure 11.

### Identification of immune-related genes associated with prognosis

We obtained 2,995 immune-related genes from 160 immune signatures curated in the TCGA pan-cancer immune landscape project [43], which was based on 11 immune relevant studies [44–54] (Supplementary Materials and Methods). We used univariate Cox proportional hazards model to examine the associations between gene expression and overall survival. Genes with false discovery rate (FDR) less than 0.05 were considered to be statistically significant and included in consensus clustering analysis. Further feature selection was conducted by using recursive feature elimination (RFE) with random forest as classifier and 10-fold cross-validation method in *R* package *caret* (version 6.0-82).

### Consensus molecular subtyping with NMF

We used nonnegative matrix factorization (NMF) to perform molecular subtyping [55, 56]. Specifically, NMF was applied to gene expression matrix *A* that contained prognostically significant immune-related genes aforementioned. Matrix *A* was factorized into 2 nonnegative matrices *W* and *H* (i.e., *A≈WH*). Repeated factorization of matrix *A* was performed and its outputs were aggregated to obtain consensus clustering of LUAD samples. The optimal number of subtypes was selected according to cophenetic, dispersion, and silhouette coefficients [57]. The consensus clustering was conducted with *R* package *NMF* (version 0.21.0) [58]. The NMF method was also used to extract mutational signatures based on the framework proposed by a previously study [59]. The *R* code was available in Supplementary Materials and Methods.

### Prediction of ICB therapy response

Potential ICB response was predicted with TIDE algorithm [15].

### Gene set enrichment analysis

The *R* package *limma* (version 3.38.3) [60] and *DESeq2* (version 1.22.2) [61] were used to calculate the differential expressed *t* statistics for microarray and RNA sequencing data. We used *t* statistic as input to *R* function *fgsea* that implemented in *fgsea* package (version 1.6.0) to perform gene set enrichment analysis (GSEA). Signaling pathways in Molecular Signatures Database (MSigDB) were used in GSEA [62].

### Identification of significantly mutated genes

Significantly mutated genes (SMGs) were identified using MutSigCV algorithm [63]. The significant enrichment of non-silent somatic mutations of a gene was measured by MutSigCV through addressing mutational context specific background mutation rates. A gene was considered an SMG if it meets these criteria: statistically significant (q < 0.1), expressed in TCGA LUAD data [64] and encyclopedia of cell lines [65], and mutation rate greater than 3%.

### Statistical analyses

*R* software 3.5.1 was applied in this study for the statistical analyses. Univariate and multivariate Cox proportional hazards model were used to analyze the association between subtypes and prognosis with *R* *survival* package. Survival curve was drawn with Kaplan-Meier method and Log-rank test was used to evaluate difference between survival curves. Association between mutation rate of SMGs and 2 subtypes was evaluated by Fisher’s exact test. Multivariate logistic regression was performed to test the association between SMGs and identified LUAD subtypes by taking into account confounding factors. The continuous and categorical variables between 2 subtypes were compared using two-sided Wilcoxon rank-sum test and chi-square test, respectively. Benjamini-Hochberg method was used to adjust for multiple hypothesis testing [61].

## Supplementary Material

Supplementary Materials

Supplementary Figures

Supplementary Tables

Supplementary Table 1
