# Supplementary material for: Analysis of immune-related signatures of lung adenocarcinoma identified two distinct subtypes: implications for immune checkpoint blockade therapy
Source: Aging (Albany NY). 2020 Feb 24;12(4):3312–39. doi: 10.18632/aging.102814 (PMC7066911; doi:10.18632/aging.102814)
Supplement: Supplementary Materials [file aging-12-102814-s002..pdf]

SUPPLEMENTARY MATERIALS

2995 immune-related genes

We obtained these 2995 immune-related genes from 160 immune signatures curated in a previous study [1], which was based on 11 immune relevant studies. Eighty-three of these 160 immune signatures were cancer immune response-related signatures, whereas the remaining 77 signatures are of general validity for immunity. These 83 signatures consisted of 68 gene sets from a earlier study [2], 9 signatures derived from TCGA gene expression data (immune metagene attractors) [3, 4], 3 signatures representing immune contexture function [5–7], and 3 signatures from a recent study [8]. The remaining 77 signatures comprised of 45 immune-cell specific signatures from 2 sources [9, 10], and 32 signatures from the ImmuneSigDB [11, 12].

Main *R* codes used in this study

```
# Nonnegative matrix factorization (NMF)
        library(NMF)
        estim.r <- nmf(gene_expression_matrix, 2:6,
        nrun = 200, method = 'brunet')
        plot(estim.r)
        consensusmap(estim.r)
        fit <- nmf(gene_expression_matrix, 2, nrun =
        200, method = "brunet")
        subtype.result <- predict(fit)
# Gene set enrichment analysis (GSEA)
        library(fgsea)
library(ggplot2)
        fgsea.result <- fgsea(pathways =
        annotation_pathways,
            stats = genes_rank_list,
            minSize = 15,
            maxSize = 500,
            nperm = 1000000)
        plotEnrichment(path[["pathway_name"]],
        genes_rank_list)
# Waterfall plot
        library(GenVisR)
waterfall(mutation_data, plotGenes = genes_to_plot,
        mainDropMut = TRUE, coverageSpace =
        30000000, clinDat = clinical_data)
# Multivariate regression model
        library(forestmodel)
library(survival)
forest_model(coxph(Surv(survival_time,
survival_end) ~ variables, related_data),
factor_separate_line = T)
forest_model(glm(categorical variable ~ variables,
binomial(), related_data), factor_separate_line = T))
```

REFERENCES

1. Thorsson V, Gibbs DL, Brown SD, Wolf D, Bortone DS, Ou Yang TH, Porta-Pardo E, Gao GF, Plaisier CL, Eddy JA, Ziv E, Culhane AC, Paull EO, et al. The Immune Landscape of Cancer. Immunity. 2018; 48:812–830.e14. https://doi.org/10.1016/j.immuni.2018.03.023 PMID:29628290

2. Wolf DM, Lenburg ME, Yau C, Boudreau A, van 't Veer LJ. Gene co-expression modules as clinically relevant hallmarks of breast cancer diversity. PLoS One. 2014; 9:e88309. https://doi.org/10.1371/journal.pone.0088309 PMID:24516633

3. Cheng WY, Ou Yang TH, Anastassiou D. Biomolecular events in cancer revealed by attractor metagenes. PLOS Comput Biol. 2013; 9:e1002920. https://doi.org/10.1371/journal.pcbi.1002920 PMID:23468608

4. Cheng WY, Ou Yang TH, Anastassiou D. Development of a prognostic model for breast cancer survival in an open challenge environment. Sci Transl Med. 2013; 5:181ra50. https://doi.org/10.1126/scitranslmed.3005974 PMID:23596202

5. Bedognetti D, Hendrickx W, Ceccarelli M, Miller LD, Seliger B. Disentangling the relationship between tumor genetic programs and immune responsiveness. Curr Opin Immunol. 2016; 39:150–58. https://doi.org/10.1016/j.coi.2016.02.001 PMID:26967649

6. Galon J, Angell HK, Bedognetti D, Marincola FM. The continuum of cancer immunosurveillance: prognostic, predictive, and mechanistic signatures. Immunity. 2013; 39:11–26. https://doi.org/10.1016/j.immuni.2013.07.008 PMID:23890060

7. Hendrickx W, Simeone I, Anjum S, Mokrab Y, Bertucci F, Finetti P, Curigliano G, Seliger B, Cerulo L, Tomei S, Delogu LG, Maccalli C, Wang E, et al. Identification of genetic determinants of breast cancer immune phenotypes by integrative genome-scale analysis. OncoImmunology. 2017; 6:e1253654. https://doi.org/10.1080/2162402X.2016.1253654 PMID:28344865

8. Şenbabaoğlu Y, Gejman RS, Winer AG, Liu M, Van Allen EM, de Velasco G, Miao D, Ostrovnaya I, Drill E, Luna A, Weinhold N, Lee W, Manley BJ, et al. Tumor immune microenvironment characterization in clear cell renal

cell carcinoma identifies prognostic and immunotherapeutically relevant messenger RNA signatures. Genome Biol. 2016; 17:231.
https://doi.org/10.1186/s13059-016-1092-z
PMID:27855702

9. Gentles AJ, Newman AM, Liu CL, Bratman SV, Feng W, Kim D, Nair VS, Xu Y, Khuong A, Hoang CD, Diehn M, West RB, Plevritis SK, Alizadeh AA. The prognostic landscape of genes and infiltrating immune cells across human cancers. Nat Med. 2015; 21:938–45.
https://doi.org/10.1038/nm.3909
PMID:26193342

10. Bindea G, Mlecnik B, Tosolini M, Kirilovsky A, Waldner M, Obenauf AC, Angell H, Fredriksen T, Lafontaine L, Berger A, Bruneval P, Fridman WH, Becker C, et al. Spatiotemporal dynamics of intratumoral immune cells reveal the immune landscape in human cancer. Immunity. 2013; 39:782–95.
https://doi.org/10.1016/j.immuni.2013.10.003
PMID:24138885

11. Godec J, Tan Y, Liberzon A, Tamayo P, Bhattacharya S, Butte AJ, Mesirov JP, Haining WN. Compendium of Immune Signatures Identifies Conserved and Species-Specific Biology in Response to Inflammation. Immunity. 2016; 44:194–206.
https://doi.org/10.1016/j.immuni.2015.12.006
PMID:26795250

12. Subramanian A, Tamayo P, Mootha VK, Mukherjee S, Ebert BL, Gillette MA, Paulovich A, Pomeroy SL, Golub TR, Lander ES, Mesirov JP. Gene set enrichment analysis: a knowledge-based approach for interpreting genome-wide expression profiles. Proc Natl Acad Sci USA. 2005; 102:15545–50.
https://doi.org/10.1073/pnas.0506580102
PMID:16199517
