# Supplementary material for: Analysis of immune-related signatures of lung adenocarcinoma identified two distinct subtypes: implications for immune checkpoint blockade therapy
Source: Aging (Albany NY). 2020 Feb 24;12(4):3312–39. doi: 10.18632/aging.102814 (PMC7066911; doi:10.18632/aging.102814)
Supplement: Supplementary Figures [file aging-12-102814-s004..pdf]

SUPPLEMENTARY FIGURES

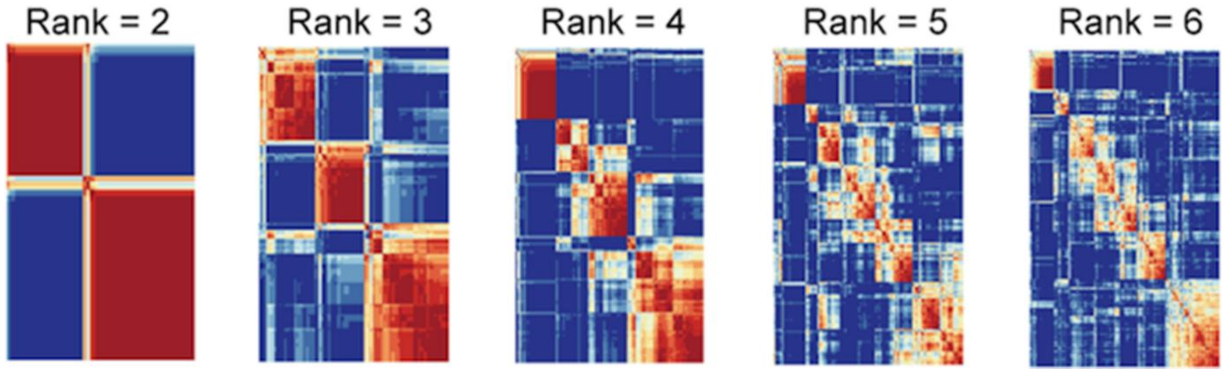

Supplementary Figure 1. Heatmap representation of NMF clustering.

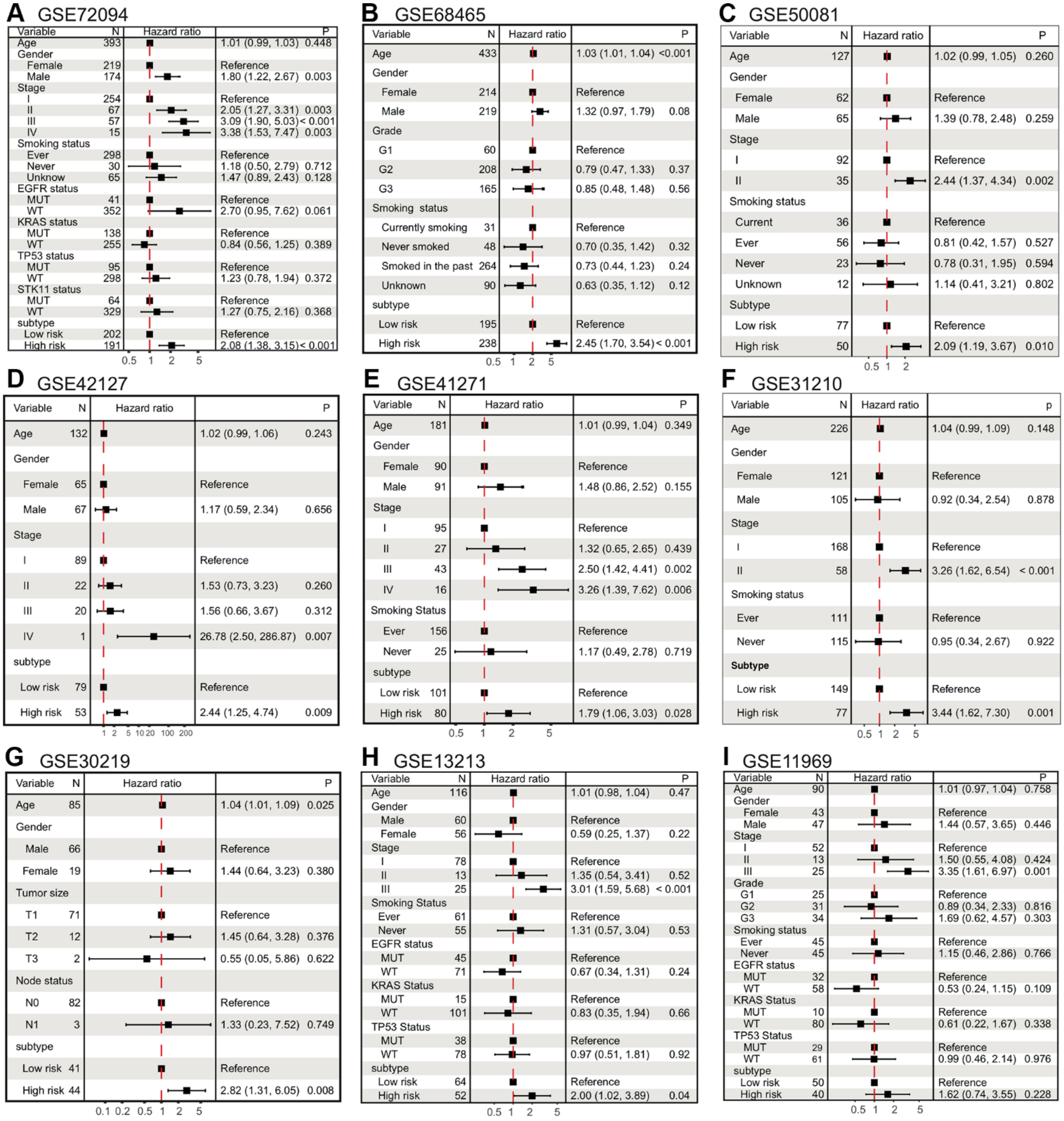

Supplementary Figure 2. Prognostic significance of high-risk versus low-risk subtypes using multivariate Cox model in 9 validation cohorts of (**A**) GSE72094, (**B**) GSE68465, (**C**) GSE50081, (**D**) GSE42127, (**E**) GSE41271, (**F**) GSE31210, (**G**) GSE30219, (**H**) GSE13213, and (**I**) GSE11969

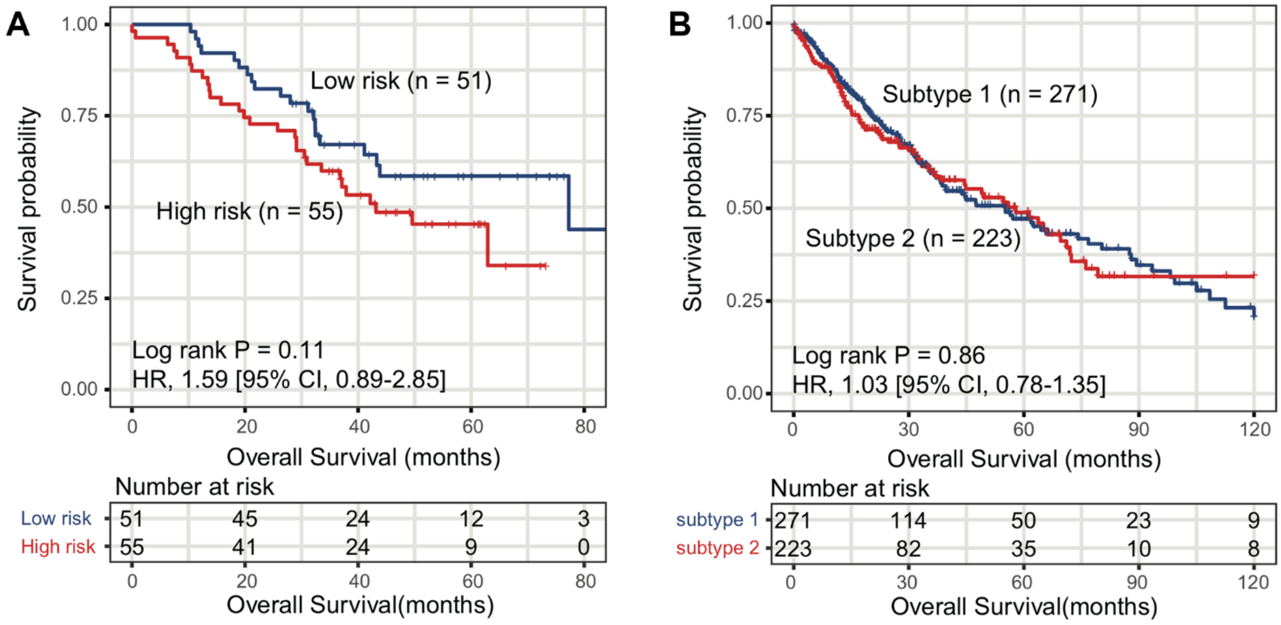

Supplementary Figure 3. (**A**) Kaplan-Meier plot of identified 2 subtypes in GSE81089 dataset. (**B**) Kaplan-Meier plot of identified TCGA LUSC subtypes.

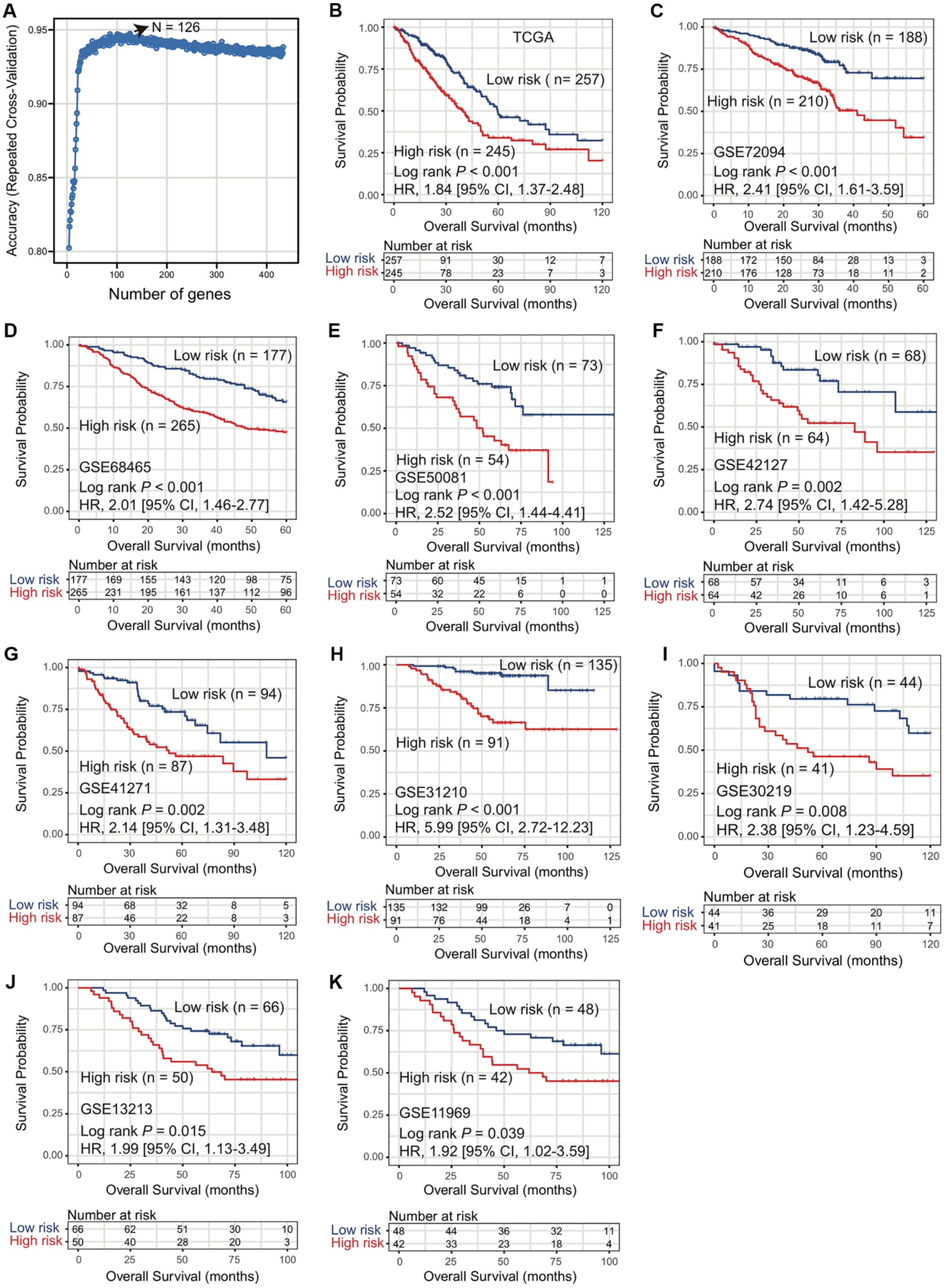

Supplementary Figure 4. (**A**) Relation between classification accuracy and selected genes via recursive feature elimination algorithm. (**B–K**) Keplan-Meier plot of identified subtypes using 126 genes in TCGA and 9 validation cohorts.

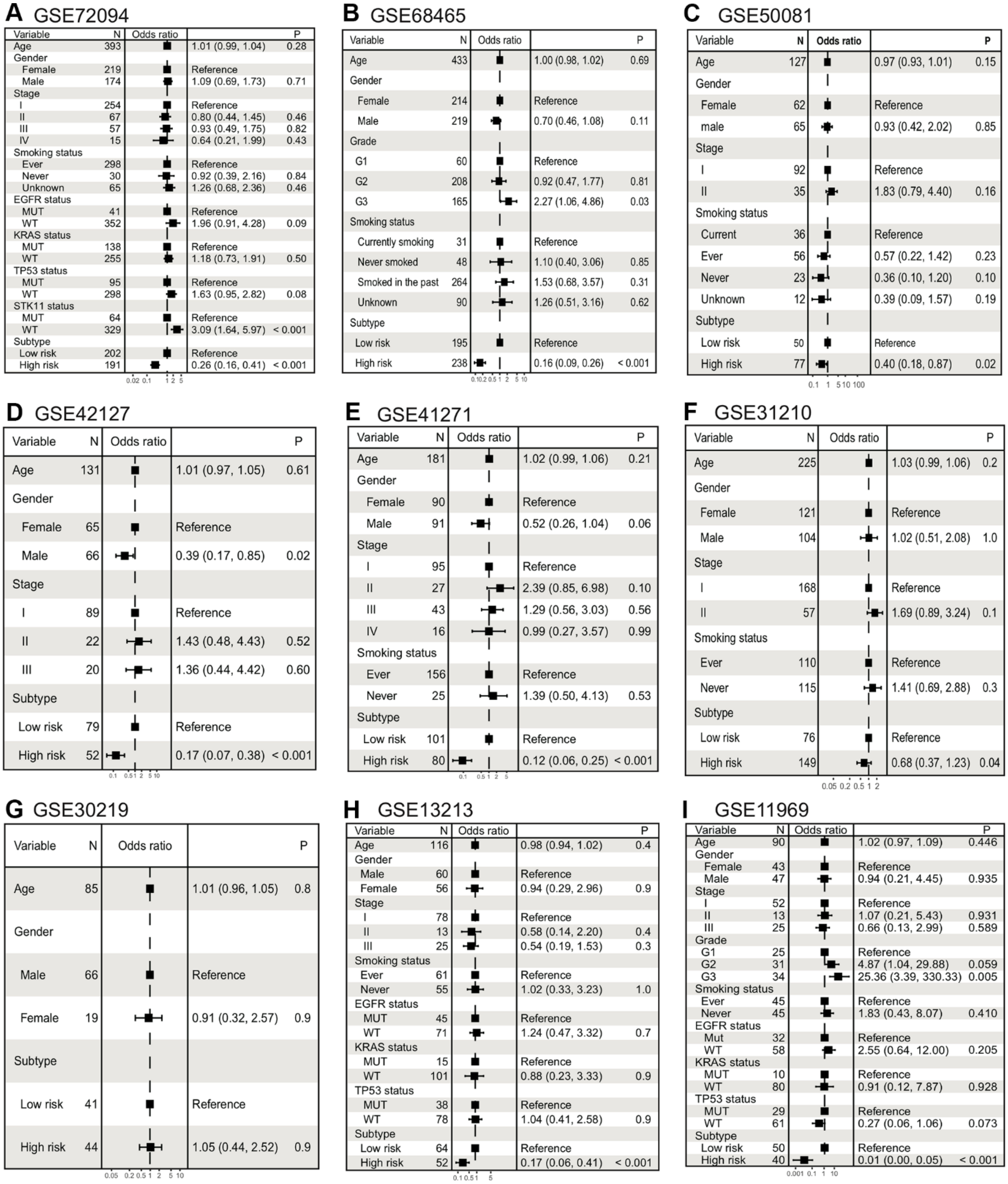

Supplementary Figure 5. Association of identified LUAD subtypes with TIDE socre using multivariate logistic analysis in 9 validation cohorts of (**A**) GSE72094, (**B**) GSE68465, (**C**) GSE50081, (**D**) GSE42127, (**E**) GSE41271, (**F**) GSE31210, (**G**) GSE30219, (**H**) GSE13213, and (**I**) GSE11969.

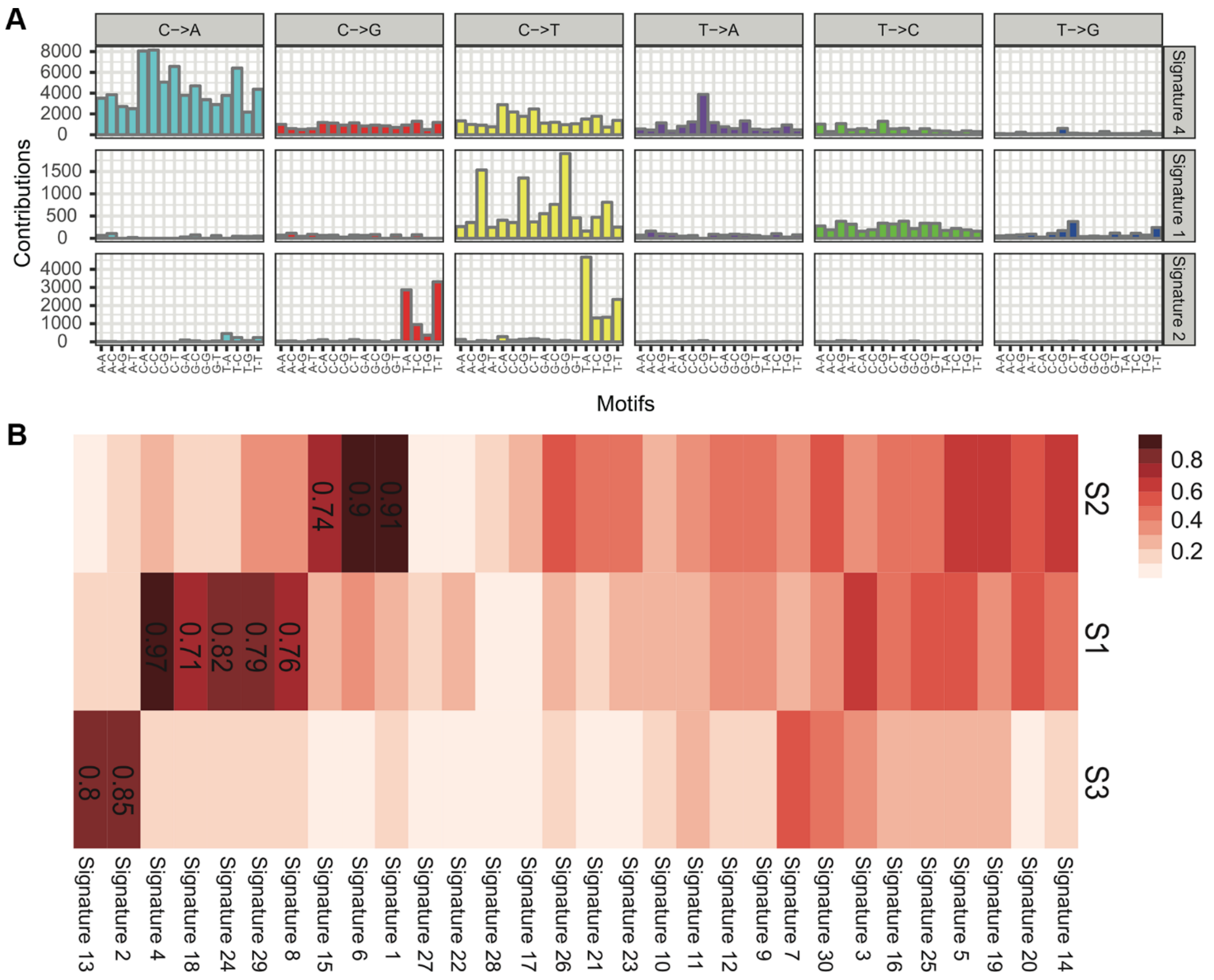

Supplementary Figure 6. (**A**) Mutational signatures extracted from TCGA LUAD cohort and (**B**) their cosine similarity with COSMIC mutational signatures.

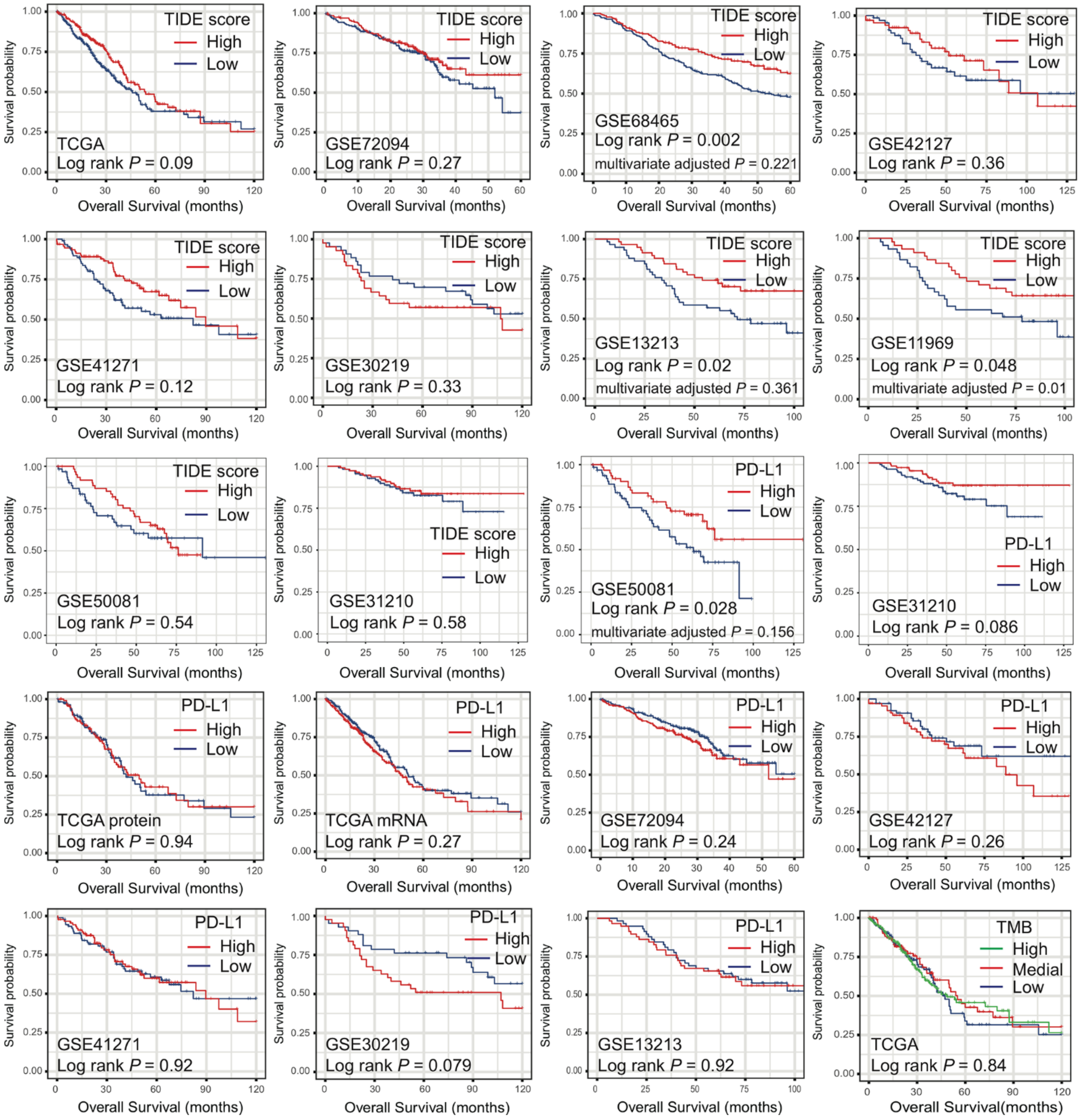

Supplementary Figure 7. Kaplan-Meier plots with respect to TIDE score, *PD-L1* expression, and TMB in TCGA and 9 validation cohorts.

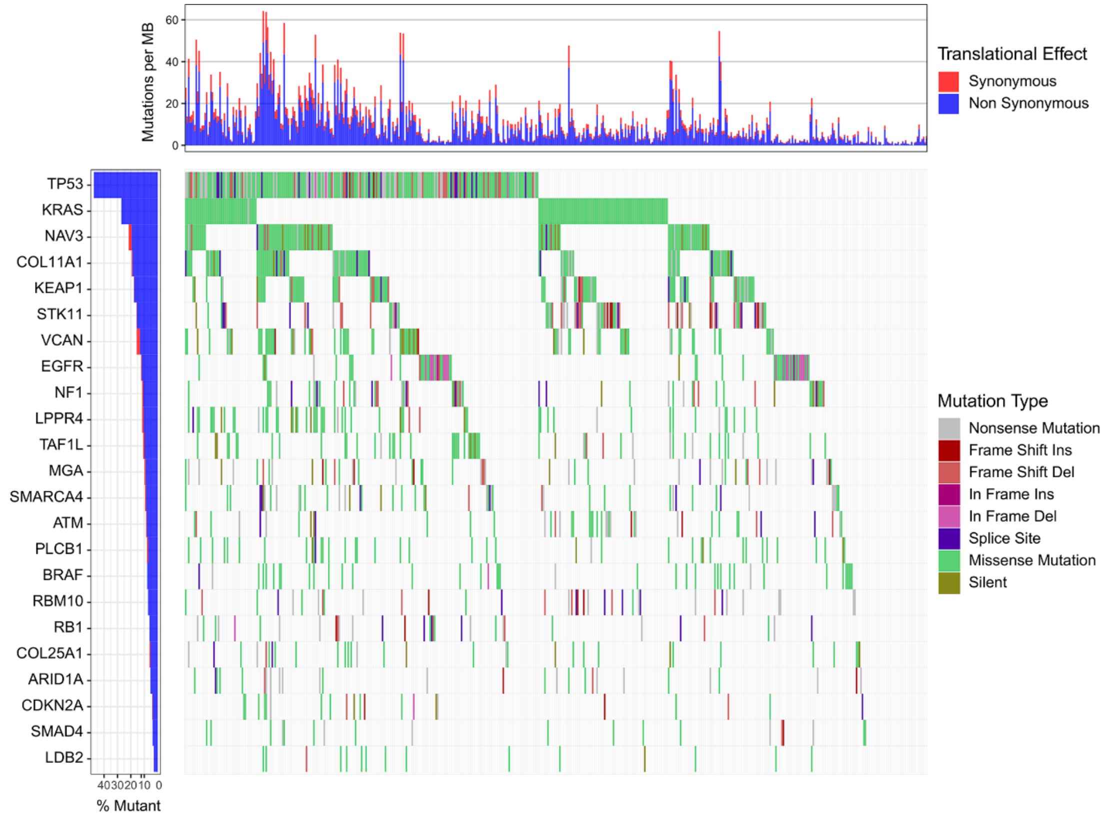

Supplementary Figure 8. Mutational landscape of SMGs in TCGA LUAD cohort.

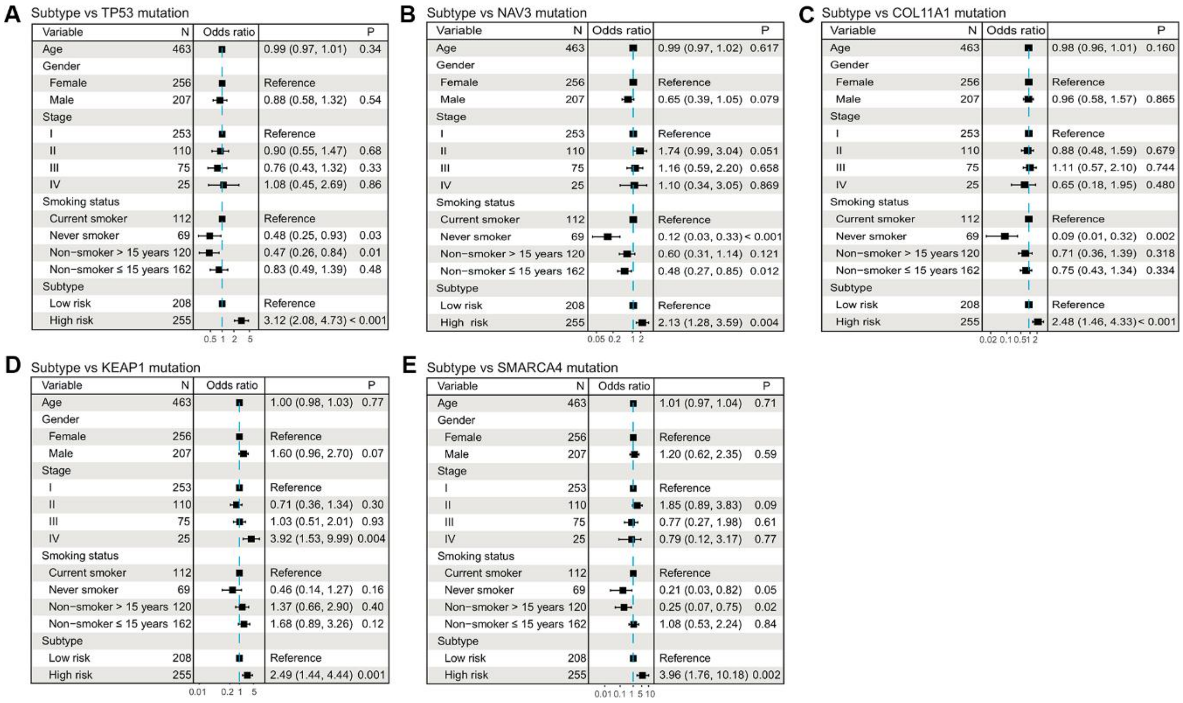

Supplementary Figure 9. Associations between mutations in *TP53*, *NAV3*, *CLO11A1*, *KEAP1* and *SMARCA4* and identified 2 LUAD subtypes using multivariate logistic analysis.

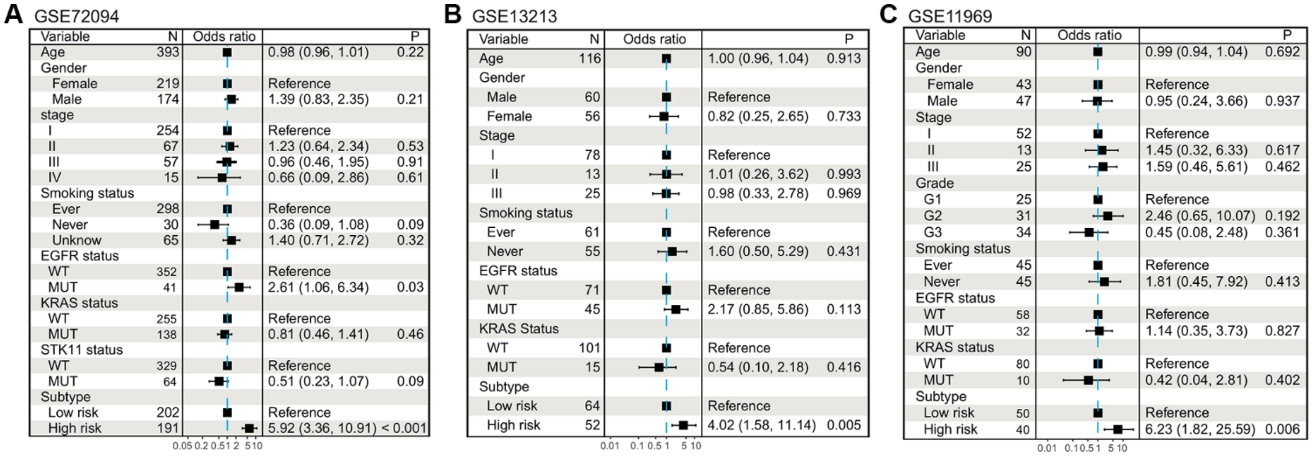

Supplementary Figure 10. Associations of *TP53* mutation with high-risk subtype in (**A**) GSE72094, (**B**) GSE13213 and (**C**) GSE11969.

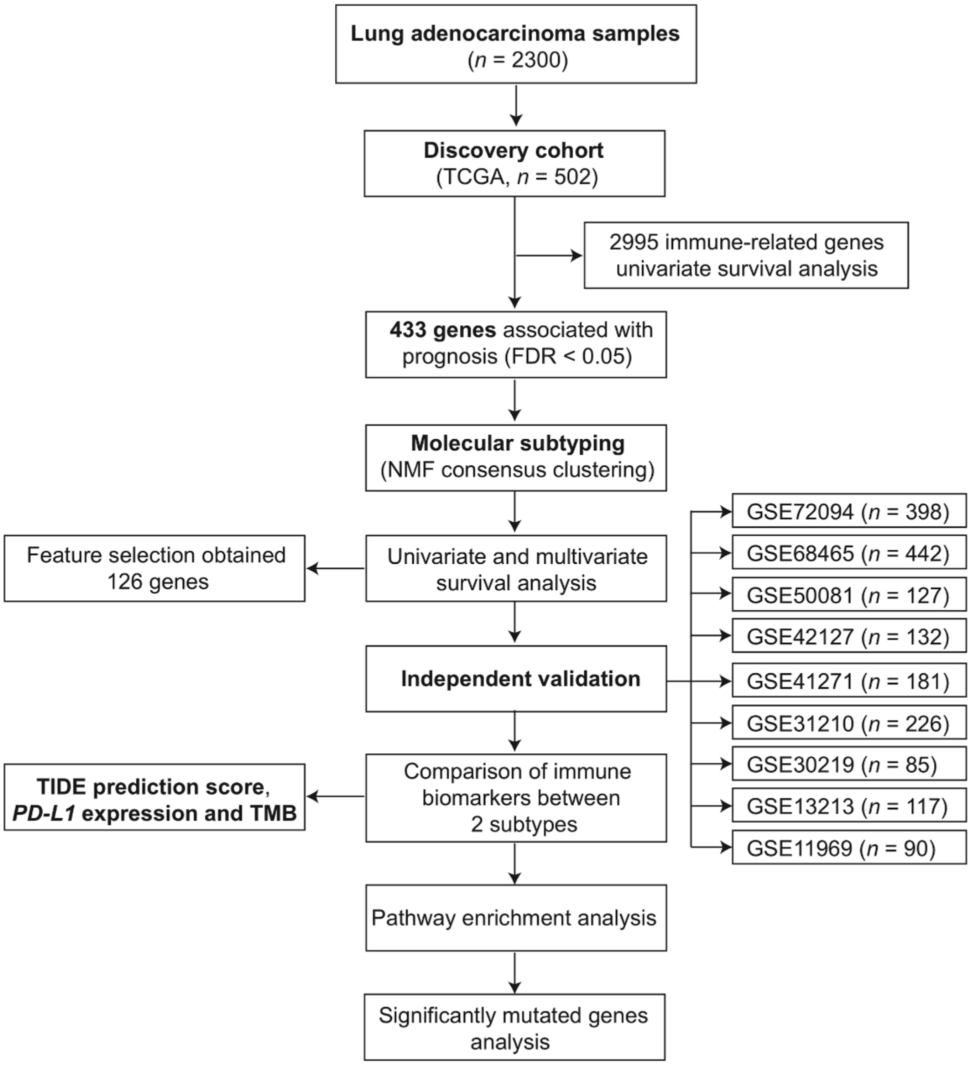

Supplementary Figure 11. Flow chart of our study. TCGA and 9 public LUAD cohorts containing 2300 samples were included to peroform relevant analyses.
