# Supplementary material for: Analysis of immune-related signatures of lung adenocarcinoma identified two distinct subtypes: implications for immune checkpoint blockade therapy
Source: Aging (Albany NY). 2020 Feb 24;12(4):3312–39. doi: 10.18632/aging.102814 (PMC7066911; doi:10.18632/aging.102814)
Supplement: Supplementary Tables [file aging-12-102814-s003..pdf]

SUPPLEMENTARY TABLES

Please browse Full Text version to see the data of Supplementary Table 1

Supplementary Table 1. 433 genes with FDR less than 0.05 in univariate survival analysis.

Supplementary Table 2. 126 genes were obtained through RFE features selection.

Gene	Entrezid	Gene	Entrezid	Gene	Entrezid	Gene	Entrezid
TSPAN32	10077	NCR3	259197	MCM10	55388	CCNB1	891
RASGRP2	10235	ASPM	259266	DEPDC1	55635	SELENBP1	8991
CDKN3	1033	STAP1	26228	PBK	55872	PRC1	9055
NDC80	10403	AMPD1	270	KIF15	56992	CD1B	910
CENPA	1058	HPGDS	27306	PTGDS	5730	CD1C	911
CENPE	1062	GNG7	2788	SPC25	57405	CD1D	912
RAD51AP1	10635	LINC00926	283663	DNASE2B	58511	CD1E	913
PLK4	10733	C11orf21	29125	RGS13	6003	CCNB2	9133
KIF2C	11004	RACGAP1	29127	RRM2	6241	EXO1	9156
UBE2C	11065	HLA-DOB	3112	BLK	640	CD5	921
CHEK1	1111	BIRC3	330	NCAPG	64151	AURKB	9212
ZWINT	11130	BIRC5	332	SFTPB	6439	CACNA2D2	9254
OIP5	11339	IL16	3603	SLAMF1	6504	CD19	930
FCRL1	115350	KLRB1	3820	SLC18A2	6571	MS4A1	931
CCR6	1235	KIF11	3832	SPIB	6689	SIGLEC6	946
ADH1A	124	LY9	4063	AURKA	6790	KIF23	9493
ADH1B	125	MAD2L1	4085	BUB1B	701	CD40LG	959
COL4A3	1285	MAL	4118	TK1	7083	ESPL1	9700
CR2	1380	MKI67	4288	CLEC3B	7123	CD79A	973
CTSG	1511	MYBL2	4605	TOP2A	7153	CD79B	974
DNASE1L3	1776	NEK2	4751	TTK	7272	ACAP1	9744
FAM129C	199786	PAX5	5079	CCR2	729230	DLGAP5	9787
SKA1	220134	NUSAP1	51203	VIPR1	7433	CDK1	983
MS4A2	2206	GTSE1	51512	DSCC1	79075	MELK	9833
FCER2	2208	PLK1	5347	ANKRD55	79722	CDC6	990
TMEM130	222865	POLE2	5427	CXorf21	80231	CDC20	991
TPX2	22974	ERCC6L	54821	TRAF3IP3	80342	KIF14	9928
FOXM1	2305	PARPBP	55010	TLR10	81793	CD302	9936
NCAPH	23397	CEP55	55165	CDC45	8318	CDC25C	995
TNFRSF13B	23495	FANCI	55215	ATP13A4	84239	HS3ST2	9956
LILRA4	23547	NEIL3	55247	FCRLA	84824		
KIF4A	24137	HJURP	55355	CCNA2	890		

Supplementary Table 3. Distinct clinical characteristics between 2 LUAD subtypes.

Characteristics	Low risk (*n*=210)	High risk (*n*=256)	*P*
Age (years)	66.6±9.1	64.2±10.6	0.125
Gender			
Female	137 (65.2)	120 (46.9)	<0.001
Male	73 (34.8)	136 (53.1)	
Stage			
I	139 (66.2)	115 (44.9)	<0.001
II	38 (18.1)	74 (28.9)	
III	25 (11.9)	50 (19.5)	
IV	8 (3.8)	17 (6.6)	
Smoking status			
Never smoker	39 (18.6)	31 (12.1)	<0.001
Non-smoker>15 years	69 (32.9)	52 (20.3)	
Non-smoker≤15 years	67 (31.9)	95 (37.1)	
Current smoker	35 (16.7)	78 (30.5)	

Supplementary Table 4. Summary of TCGA and 9 validation datasets included in this study.

Datasets	Platforms	Sample size
TCGA	Illumina RNAseq HTSeq	502
GSE72094	Rosetta/Merck Human RSTA Custom Affymetrix 2.0 microarray	398
GSE68465	Affymetrix Human Genome U133A Array	442
GSE50081	Affymetrix Human Genome U133 Plus 2.0 Array	127
GSE42127	Illumina HumanWG-6 v3.0 expression beadchip	132
GSE41271	Illumina HumanWG-6 v3.0 expression beadchip	181
GSE31210	Affymetrix Human Genome U133 Plus 2.0 Array	226
GSE30219	Affymetrix Human Genome U133 Plus 2.0 Array	85
GSE13213	Agilent-014850 Whole Human Genome Microarray 4x44K G4112F	117
GSE11969	Agilent Homo sapiens 21.6K custom array	90
Total		2300

Supplementary Table 5. Descriptions of clinical characteristics for LUAD patients in TCGA and 9 validation datasets.

Characteristics	TCGA	GSE72094	GSE68465	GSE50081	GSE42127	GSE41271	GSE31210	GSE30219	GSE13213	GSE11969
Number of	502	398	422	127	132	181	226	85	117	90
Age (years)	65.3 ± 9.9	69.4 ± 9.5	64.5 ± 10.1	68.7 ± 9.7	65.8 ± 10.3	64.6 ± 10.4	59.6 ± 7.4	61.5 ± 9.3	60.7 ± 10.2	61.1 ± 9.8
Gender										
Male	209	174	219	65	67	91	105	66	60	47
Female	257	219	214	62	65	90	121	19	56	43
Stage<br>I	254	254		92	89	95	168		78	52
II	112	67	-	35	22	27	58	-	13	13
III	75	57	-	0	20	43	0	-	25	25
IV	25	15	-	0	1	16	0	-	-	-
Grade										
G1			60							25
G2	-	-	208	-	-	-	-	-	-	31
G3	-	-	165	-	-	-	-	-	-	34
Smoking status<br>Never smoker	70	298	15	23		25	111		55	45
Ever smoker	-	30	94	56	-	156	115	-	61	45
Current smoker	113	-	16	36	-	-	-	-	-	-
Non-smoker>15	121	-	-	-	-	-	-	-	-	-
Non-smoker≤ 15 years	162	-	-	-	-	-	-	-	-	-
NA	-	65	90	12	-	-	-	-	-	-
