# Supplementary material for: Analysis of immune-related signatures of lung adenocarcinoma identified two distinct subtypes: implications for immune checkpoint blockade therapy
Source: Aging (Albany NY). 2020 Feb 24;12(4):3312–39. doi: 10.18632/aging.102814 (PMC7066911; doi:10.18632/aging.102814)
Supplement: Supplementary Table 1 [file aging-12-102814-s001..docx]

**Supplementary Table 1.** 433 genes with FDR less than 0.05 in univariate survival analysis.

| Gene | Entrezid | FDR | Gene | Entrezid | FDR | Gene | Entrezid | FDR |
| --- | --- | --- | --- | --- | --- | --- | --- | --- |
| CYP17A1 | 1586 | 0.000954069 | ACTN1 | 87 | 0.009966026 | CD1E | 913 | 0.025897351 |
| FLNC | 2318 | 0.000954069 | LMNB2 | 84823 | 0.010073975 | SNRPA | 6626 | 0.025897351 |
| FUT4 | 2526 | 0.000954069 | GRAMD1B | 57476 | 0.01010396 | TAF2 | 6873 | 0.026597023 |
| ITGA6 | 3655 | 0.000954069 | RAB11A | 8766 | 0.010406856 | FAM104A | 84923 | 0.026597023 |
| KRT6A | 3853 | 0.000954069 | RHOC | 389 | 0.01051817 | BACH1 | 571 | 0.026602328 |
| KRT8 | 3856 | 0.000954069 | MCM5 | 4174 | 0.01051817 | FCER2 | 2208 | 0.026847705 |
| SHC1 | 6464 | 0.000954069 | KIF2C | 11004 | 0.01051817 | MAP2K1 | 5604 | 0.026847705 |
| VDAC1 | 7416 | 0.000954069 | TLR10 | 81793 | 0.01051817 | TRAF3IP3 | 80342 | 0.026847705 |
| RGS20 | 8601 | 0.000954069 | CTNNA1 | 1495 | 0.010681697 | RALGPS2 | 55103 | 0.026862957 |
| PLEK2 | 26499 | 0.000954069 | DRP2 | 1821 | 0.010681697 | TIMP1 | 7076 | 0.02701245 |
| LINGO2 | 158038 | 0.000954069 | MCM4 | 4173 | 0.01087191 | HLA-DOB | 3112 | 0.027072943 |
| KRT6C | 286887 | 0.000954069 | MAPK6 | 5597 | 0.01089323 | NID1 | 4811 | 0.027072943 |
| PLK1 | 5347 | 0.001246868 | TMPO | 7112 | 0.011066166 | MRPS16 | 51021 | 0.027072943 |
| VEGFC | 7424 | 0.001343868 | LMNB1 | 4001 | 0.011306877 | FCRLA | 84824 | 0.027079759 |
| PKP2 | 5318 | 0.001430168 | SMAD3 | 4088 | 0.011306877 | PAK1 | 5058 | 0.027132173 |
| DLGAP5 | 9787 | 0.001430168 | RACGAP1 | 29127 | 0.011306877 | HSPA1B | 3304 | 0.027351164 |
| ECT2 | 1894 | 0.001436128 | NAA50 | 80218 | 0.011306877 | BSG | 682 | 0.027415388 |
| KRT6B | 3854 | 0.001436128 | RNPEP | 6051 | 0.011499731 | SCD | 6319 | 0.027415388 |
| LOXL2 | 4017 | 0.001436128 | NMI | 9111 | 0.011499731 | DHRS7 | 51635 | 0.027415388 |
| FOSL1 | 8061 | 0.001436128 | CDCA4 | 55038 | 0.011499731 | RMDN1 | 51115 | 0.027669448 |
| CTSL | 1514 | 0.001544707 | CD19 | 930 | 0.011578803 | OSBPL10 | 114884 | 0.027669448 |
| KIF14 | 9928 | 0.001544707 | CDC6 | 990 | 0.011578803 | DHFR | 1719 | 0.028226413 |
| SLC2A1 | 6513 | 0.001686142 | CENPA | 1058 | 0.011578803 | TJP1 | 7082 | 0.028325301 |
| CCNB1 | 891 | 0.00171359 | PGD | 5226 | 0.011578803 | MICAL3 | 57553 | 0.028347499 |
| HMMR | 3161 | 0.00171359 | PDLIM7 | 9260 | 0.011578803 | SLAMF1 | 6504 | 0.02850302 |
| ASPM | 259266 | 0.00171359 | NCAPH | 23397 | 0.011578803 | LILRA4 | 23547 | 0.02850302 |
| CCNA2 | 890 | 0.001768515 | ZWILCH | 55055 | 0.011578803 | UBE2L3 | 7332 | 0.028724699 |
| HMGA1 | 3159 | 0.001768515 | SERPINB7 | 8710 | 0.011696849 | CD1B | 910 | 0.029172281 |
| RRM2 | 6241 | 0.001768515 | MMP14 | 4323 | 0.011819673 | ADAR | 103 | 0.029314891 |
| EXO1 | 9156 | 0.001768515 | MAP2K3 | 5606 | 0.011819673 | FCRL2 | 79368 | 0.029655615 |
| PKM | 5315 | 0.00181238 | ATP8A1 | 10396 | 0.011819673 | DCBLD2 | 131566 | 0.029655615 |
| TNS4 | 84951 | 0.00181238 | ESPL1 | 9700 | 0.011983835 | CD5 | 921 | 0.029850516 |
| EHBP1 | 23301 | 0.001843297 | MELK | 9833 | 0.012047507 | MRPL37 | 51253 | 0.029998734 |
| MAP4K4 | 9448 | 0.001908214 | TNFRSF6B | 8771 | 0.01204914 | BLVRB | 645 | 0.03014453 |
| LAMC2 | 3918 | 0.001923511 | C11orf24 | 53838 | 0.01204914 | SIAH2 | 6478 | 0.030586258 |
| PRC1 | 9055 | 0.001923511 | TMEM130 | 222865 | 0.01204914 | LINC00926 | 283663 | 0.031122546 |
| ERLIN1 | 10613 | 0.001923511 | COL4A1 | 1282 | 0.012164828 | NLN | 57486 | 0.031440654 |
| ANP32B | 10541 | 0.001947745 | PLAUR | 5329 | 0.012331435 | COL1A1 | 1277 | 0.031969128 |
| HJURP | 55355 | 0.001947745 | BCL2L1 | 598 | 0.012925838 | COL4A3 | 1285 | 0.031969128 |
| SMS | 6611 | 0.002184859 | HNRNPA2B1 | 3181 | 0.012925838 | TGFBR1 | 7046 | 0.032133368 |
| KYNU | 8942 | 0.002184859 | HSPA1A | 3303 | 0.012925838 | DSP | 1832 | 0.032217766 |
| NEIL3 | 55247 | 0.002184859 | TOMM40 | 10452 | 0.012925838 | PSMD4 | 5710 | 0.032217766 |
| CDKN3 | 1033 | 0.002186332 | PLEKHG3 | 26030 | 0.012925838 | GGH | 8836 | 0.032217766 |
| TPX2 | 22974 | 0.002186332 | ERCC6L | 54821 | 0.012925838 | PARP1 | 142 | 0.032816082 |
| TGIF1 | 7050 | 0.002321019 | ENO1 | 2023 | 0.012977141 | OAS3 | 4940 | 0.032816082 |
| NEK2 | 4751 | 0.002461469 | TPM3 | 7170 | 0.013098096 | RUVBL1 | 8607 | 0.033644774 |
| LAMB1 | 3912 | 0.002582341 | SNRPA1 | 6627 | 0.013510592 | RTN3 | 10313 | 0.034619422 |
| MYO1E | 4643 | 0.002582341 | PLSCR1 | 5359 | 0.013530346 | TIPIN | 54962 | 0.035134198 |
| AKAP12 | 9590 | 0.002582341 | TSPAN32 | 10077 | 0.013562469 | PARPBP | 55010 | 0.035134198 |
| OIP5 | 11339 | 0.002582341 | IL2 | 3558 | 0.013644533 | CTSG | 1511 | 0.035316973 |
| PXN | 5829 | 0.002677536 | UBE2C | 11065 | 0.013775277 | HPCAL1 | 3241 | 0.035470587 |
| JAG1 | 182 | 0.002696042 | RND3 | 390 | 0.013872246 | DUSP5 | 1847 | 0.035615107 |
| LPGAT1 | 9926 | 0.002696042 | PAK2 | 5062 | 0.013890802 | RPS2 | 6187 | 0.035615107 |
| FOXM1 | 2305 | 0.002725746 | C11orf21 | 29125 | 0.013950983 | NFE2L3 | 9603 | 0.035615107 |
| GNG7 | 2788 | 0.002725746 | TNFRSF13B | 23495 | 0.014111175 | SLC39A14 | 23516 | 0.03578486 |
| PFKP | 5214 | 0.003001652 | S100A11 | 6282 | 0.014331485 | FERMT2 | 10979 | 0.035891323 |
| TPI1 | 7167 | 0.003001652 | CLEC3B | 7123 | 0.014331485 | NUP107 | 57122 | 0.035891323 |
| ADM | 133 | 0.003084187 | VIPR1 | 7433 | 0.014331485 | NUDT1 | 4521 | 0.036221036 |
| CDC25C | 995 | 0.003162828 | FARSB | 10056 | 0.014331485 | CXorf21 | 80231 | 0.036221036 |
| CHEK1 | 1111 | 0.003178275 | ZWINT | 11130 | 0.014331485 | ATP13A4 | 84239 | 0.036221036 |
| SMOX | 54498 | 0.003178275 | NUDT15 | 55270 | 0.014331485 | IMP4 | 92856 | 0.036221036 |
| PSMD2 | 5708 | 0.003326959 | C19orf48 | 84798 | 0.01453333 | YME1L1 | 10730 | 0.037135697 |
| XCR1 | 2829 | 0.003342366 | MCM10 | 55388 | 0.014933465 | PAX5 | 5079 | 0.037251137 |
| SLC16A1 | 6566 | 0.003342366 | ANP32E | 81611 | 0.014933465 | PPIC | 5480 | 0.038252431 |
| SPC25 | 57405 | 0.003342366 | EFHD2 | 79180 | 0.014971629 | KIR2DL3 | 3804 | 0.038395545 |
| MKI67 | 4288 | 0.00336399 | CCR2 | 729230 | 0.014971629 | LPCAT4 | 254531 | 0.038395545 |
| RHOF | 54509 | 0.003454214 | MAL | 4118 | 0.01514394 | NOP16 | 51491 | 0.038404838 |
| APOL1 | 8542 | 0.003487711 | KIAA1324 | 57535 | 0.01514394 | DNASE1L3 | 1776 | 0.038800311 |
| KIF4A | 24137 | 0.003530385 | COPS8 | 10920 | 0.015159317 | UMPS | 7372 | 0.038800311 |
| SHCBP1 | 79801 | 0.003530385 | PPP3CA | 5530 | 0.015233555 | CD79A | 973 | 0.039139257 |
| CDK1 | 983 | 0.003913595 | HSPA5 | 3309 | 0.015340963 | LDHB | 3945 | 0.039588158 |
| KIF11 | 3832 | 0.003913595 | ARHGDIA | 396 | 0.01544147 | RGS13 | 6003 | 0.039759592 |
| WDHD1 | 11169 | 0.003913595 | EGLN3 | 112399 | 0.01544147 | PHKA1 | 5255 | 0.040086734 |
| SFTPB | 6439 | 0.003938287 | VPS18 | 57617 | 0.015463088 | RELB | 5971 | 0.040086734 |
| KIF18A | 81930 | 0.003938287 | DSCC1 | 79075 | 0.015614829 | BRIP1 | 83990 | 0.040086734 |
| XRCC6 | 2547 | 0.004001742 | DNAJC10 | 54431 | 0.016289614 | CR2 | 1380 | 0.040192419 |
| CENPF | 1063 | 0.004084557 | PYGL | 5836 | 0.016411453 | RAD54B | 25788 | 0.040192419 |
| LAMC1 | 3915 | 0.004226514 | TOP2A | 7153 | 0.01710411 | DNASE2B | 58511 | 0.040192419 |
| TK1 | 7083 | 0.004226514 | TUBA4A | 7277 | 0.01710411 | CSNK2A1 | 1457 | 0.040847185 |
| CACNA2D2 | 9254 | 0.004226514 | CENPN | 55839 | 0.01710411 | JUP | 3728 | 0.040847185 |
| NDC80 | 10403 | 0.004226514 | PLK4 | 10733 | 0.017165425 | PDHX | 8050 | 0.040847185 |
| PAICS | 10606 | 0.004226514 | CSNK1A1 | 1452 | 0.017224589 | SOD2 | 6648 | 0.041148028 |
| DEPDC1 | 55635 | 0.004226514 | BLK | 640 | 0.017291684 | KRR1 | 11103 | 0.041148028 |
| PBK | 55872 | 0.004226514 | SIGLEC6 | 946 | 0.017291684 | PTGDS | 5730 | 0.041249118 |
| FCRL1 | 115350 | 0.004226514 | AURKB | 9212 | 0.01732247 | PITPNC1 | 26207 | 0.041652518 |
| STAP1 | 26228 | 0.004303086 | NCAPG2 | 54892 | 0.017551823 | CYFIP1 | 23191 | 0.041943834 |
| PGAM1 | 5223 | 0.004343967 | FBXO28 | 23219 | 0.017595079 | PTPN12 | 5782 | 0.04207023 |
| PMEPA1 | 56937 | 0.004343967 | FUBP3 | 8939 | 0.017641663 | CD79B | 974 | 0.042194065 |
| PABPC1 | 26986 | 0.004459156 | CISH | 1154 | 0.017869865 | COL6A1 | 1291 | 0.042194065 |
| KIF23 | 9493 | 0.004478674 | PSMD14 | 10213 | 0.018244942 | GJB5 | 2709 | 0.042194065 |
| H2AFZ | 3015 | 0.00452547 | TM4SF1 | 4071 | 0.01839594 | DNAJB1 | 3337 | 0.042194065 |
| LYAR | 55646 | 0.004568678 | LY9 | 4063 | 0.018507227 | CHMP2A | 27243 | 0.042194065 |
| CCNB2 | 9133 | 0.004676963 | EPHA2 | 1969 | 0.018594398 | PICALM | 8301 | 0.042317724 |
| CENPE | 1062 | 0.004767585 | TTK | 7272 | 0.018819425 | BAK1 | 578 | 0.042490063 |
| TNFRSF1A | 7132 | 0.004767585 | HPGDS | 27306 | 0.018819425 | MXD1 | 4084 | 0.042490063 |
| EIF4G1 | 1981 | 0.004772564 | CDC45 | 8318 | 0.018935218 | RASGRP2 | 10235 | 0.042490063 |
| CEP55 | 55165 | 0.004772564 | CCT5 | 22948 | 0.018935218 | RAD51AP1 | 10635 | 0.042490063 |
| TACC3 | 10460 | 0.004832608 | CHPF | 79586 | 0.018935218 | CKAP2 | 26586 | 0.042490063 |
| ANGPTL4 | 51129 | 0.00505869 | CCR6 | 1235 | 0.019157342 | BCCIP | 56647 | 0.042490063 |
| TEAD4 | 7004 | 0.005118938 | CD1C | 911 | 0.019336832 | AMFR | 267 | 0.042523535 |
| CYCS | 54205 | 0.005275205 | SERPINE1 | 5054 | 0.019412237 | FBXO5 | 26271 | 0.042523535 |
| NCAPG | 64151 | 0.005414548 | TNNC2 | 7125 | 0.019412237 | AIMP2 | 7965 | 0.042548346 |
| GTSE1 | 51512 | 0.005771753 | FUBP1 | 8880 | 0.019602702 | PDXK | 8566 | 0.042975212 |
| CAMSAP2 | 23271 | 0.005782945 | PDLIM5 | 10611 | 0.019696707 | CD302 | 9936 | 0.043147371 |
| MS4A1 | 931 | 0.005862512 | SLC18A2 | 6571 | 0.020202123 | MS4A2 | 2206 | 0.043243937 |
| MTHFD1 | 4522 | 0.005862512 | YBX3 | 8531 | 0.020259747 | IGFBP3 | 3486 | 0.043243937 |
| PA2G4 | 5036 | 0.005862512 | CDK5R1 | 8851 | 0.020259747 | MAP4 | 4134 | 0.043243937 |
| RIPK2 | 8767 | 0.005862512 | ACTL6A | 86 | 0.020336331 | ZNF598 | 90850 | 0.043281703 |
| FKBP9 | 11328 | 0.005862512 | FDPS | 2224 | 0.020336331 | EEF1E1 | 9521 | 0.043982581 |
| MAD2L1 | 4085 | 0.005939204 | VCL | 7414 | 0.020336331 | MAFF | 23764 | 0.043982581 |
| PFN1 | 5216 | 0.005939204 | PPP6R1 | 22870 | 0.020336331 | ADH1B | 125 | 0.043987156 |
| PTTG1 | 9232 | 0.006388208 | TMEM106B | 54664 | 0.020336331 | DHX32 | 55760 | 0.043987156 |
| FANCI | 55215 | 0.006388208 | MSMO1 | 6307 | 0.020372297 | G6PC | 2538 | 0.044722086 |
| CDC20 | 991 | 0.006554284 | ATAD2 | 29028 | 0.020413347 | IL11 | 3589 | 0.04477838 |
| SEMA4B | 10509 | 0.006554284 | SLC7A5 | 8140 | 0.020961116 | MARCKS | 4082 | 0.044991361 |
| BIRC3 | 330 | 0.006559509 | WWP1 | 11059 | 0.020961116 | MAPRE1 | 22919 | 0.044991361 |
| CD40LG | 959 | 0.006559509 | WSB2 | 55884 | 0.020961116 | ZIC2 | 7546 | 0.045315112 |
| PYGB | 5834 | 0.006580602 | CEBPB | 1051 | 0.021001213 | SDCBP | 6386 | 0.045416073 |
| BUB1B | 701 | 0.006744403 | RTN4 | 57142 | 0.021055347 | SQLE | 6713 | 0.045506829 |
| AURKA | 6790 | 0.006804775 | TUBB6 | 84617 | 0.021401309 | GINS1 | 9837 | 0.045506829 |
| ZNF185 | 7739 | 0.006924006 | MYBL2 | 4605 | 0.021582824 | KIF15 | 56992 | 0.045506829 |
| ZC3H15 | 55854 | 0.0069846 | NLRP1 | 22861 | 0.021776027 | EPHX1 | 2052 | 0.045730511 |
| S100A10 | 6281 | 0.007018285 | HIST1H2BK | 85236 | 0.021776027 | MET | 4233 | 0.045730511 |
| FEN1 | 2237 | 0.007503949 | HS3ST2 | 9956 | 0.022263942 | NME1 | 4830 | 0.045730511 |
| KLRB1 | 3820 | 0.00762902 | S100P | 6286 | 0.022475226 | RAD21 | 5885 | 0.045730511 |
| IL1R2 | 7850 | 0.00762902 | UAP1 | 6675 | 0.022475226 | TRA2B | 6434 | 0.045730511 |
| NCAPD2 | 9918 | 0.00762902 | EFNA5 | 1946 | 0.022706436 | SPIB | 6689 | 0.045730511 |
| PSMD12 | 5718 | 0.007691617 | HIST1H2BC | 8347 | 0.023564606 | TPM2 | 7169 | 0.045730511 |
| RAB10 | 10890 | 0.007691617 | LAMA3 | 3909 | 0.02383281 | CAV1 | 857 | 0.045816793 |
| BIRC5 | 332 | 0.007857235 | APOL2 | 23780 | 0.02383281 | ANKRD55 | 79722 | 0.046077 |
| IARS2 | 55699 | 0.00802021 | COL1A2 | 1278 | 0.023977861 | JAK1 | 3716 | 0.046111412 |
| RAC1 | 5879 | 0.008184234 | POLE2 | 5427 | 0.024424503 | ACAP1 | 9744 | 0.046831828 |
| ITGA5 | 3678 | 0.008209689 | MAGOHB | 55110 | 0.024677993 | CD1D | 912 | 0.047110127 |
| TAX1BP3 | 30851 | 0.008261659 | MRPL12 | 6182 | 0.024829411 | IL16 | 3603 | 0.047266518 |
| UQCRB | 7381 | 0.008298907 | ADAM9 | 8754 | 0.024858928 | SRM | 6723 | 0.047439165 |
| DUT | 1854 | 0.008449012 | HMGB1 | 3146 | 0.025026159 | SELENBP1 | 8991 | 0.04899826 |
| SMC2 | 10592 | 0.008449012 | SAMD9 | 54809 | 0.025360304 | CCDC91 | 55297 | 0.049152201 |
| NUSAP1 | 51203 | 0.00857285 | ADA | 100 | 0.025598425 | APBB2 | 323 | 0.04923467 |
| ERRFI1 | 54206 | 0.00857285 | FAM129C | 199786 | 0.025598425 | ADH1A | 124 | 0.049533177 |
| PGM2 | 55276 | 0.008586277 | NUP88 | 4927 | 0.025662321 | SNRPD1 | 6632 | 0.049557883 |
| SNRPE | 6635 | 0.008674691 | NCR3 | 259197 | 0.025662321 | LSM4 | 25804 | 0.049748066 |
| HNRNPR | 10236 | 0.008674691 | TAP2 | 6891 | 0.025683946 | CD160 | 11126 | 0.049757515 |
| CPSF2 | 53981 | 0.009282509 | ZNF207 | 7756 | 0.025683946 | P4HA2 | 8974 | 0.049893406 |
| PLOD2 | 5352 | 0.009605655 | VTA1 | 51534 | 0.025683946 |  |  |  |
| SKA1 | 220134 | 0.009882868 | AMPD1 | 270 | 0.025897351 |  |  |  |
